# Modeling learner-controlled mental model learning processes by a second-order adaptive network model

**DOI:** 10.1371/journal.pone.0255503

**Published:** 2021-08-24

**Authors:** Rajesh Bhalwankar, Jan Treur

**Affiliations:** 1 Work and Social Psychology Department, Maastricht University, Maastricht, Netherlands; 2 Social AI Group, Vrije Universiteit Amsterdam, Amsterdam, Netherlands; Unviersity of Burgundy, FRANCE

## Abstract

Learning knowledge or skills usually is considered to be based on the formation of an adequate internal mental model as a specific type of mental network. The learning process for such a mental model conceptualised as a mental network, is a form of (first-order) mental network adaptation. Such learning often integrates learning by observation and learning by instruction. For an effective learning process, an appropriate timing of these different elements is crucial. By controlling the timing of them, the mental network adaptation process becomes adaptive itself, which is called second-order mental network adaptation. In this paper, a second-order adaptive mental network model is proposed addressing this. The first-order adaptation process models the learning process of mental models and the second-order adaptation process controls the timing of the elements of this learning process. It is illustrated by a case study for the learner-controlled mental model learning in the context of driving a car. Here the learner is in control of the integration of learning by observation and learning by instruction.

## 1 Introduction

To describe the mental processes involving learning and problem solving in humans, often mental models are used, e.g., [1–16]. As a specific case, mental models of devices and their usage are formed to be able to adequately use these devices, e.g., [17, 18]. It is an interesting challenge to determine how mental models are formed or learnt, and how to control such learning processes. Computational models which represent such processes are almost absent, e.g., [19–21]. One exception is [8] in which a production rule modeling format is used to simulate students’ construction of energy models for learning physics. In general, however, research into how mental models develop or are learnt and how that is controlled, is hard to find.

The current paper proposes such a computational model for mental model learning and its control, based on multi-order adaptive network-oriented modeling [22, 23]. It is illustrated by a case study for learning how a car works and how to drive it. A driver’s mental model and how it can be learnt in an effective manner can be a basis for the designing virtual pedagogical agents, and for support of a driver by adaptive automation in a car.

Network-oriented modeling for adaptive networks [22–24] is an effective approach to model the adaptive mental processes as an adaptive interplay of mental states. Here the connections between the mental states change based on specific adaptation principles such as Hebbian learning [25]. Learning mental models involves such adaptation, but it also involves controlling this learning; the latter is a form of second-order adaptation. The network-oriented modeling approach from [22–24] covers such multi-order adaptive processes.

Then the picture is that a mental model can be modeled as a base network and learning the mental model can be modeled as (first-order) adaptation of this base network. In addition, the controlling of this learning process can be modeled as second-order adaptation, which adapts the first-order adaptation. In this way, a three-level second-order adaptive network architecture for mental model development is obtained. It is illustrated here for learning a mental model, by a learner-controlled interplay of observational and instructional learning in a case study for learning how a car works and how to drive it.

Part of this work was addressed in a preliminary form in [26]. However, the current paper extends this by more than 110%. In the current paper, in particular, (1) the description of the computational model and its background (Sections 2 and 4) has been extended much so that now a much more detailed design description is provided and (2) also an extensive analysis of equilibria of the model is now described (Section 6) which has been conducted to obtain a more solid basis for the implemented model by verification (Section 6 on equilibrium analysis of the model in the current paper is completely new). All this was not addressed in [26].

In the paper, Section 2 presents a brief literature overview. In Section 3, the design of the proposed second-order adaptive network architecture is presented, addressing the controlled interplay between observational and instructional learning of mental models. Section 4 presents a refinement of this architecture, addressing the case study of learner-controlled integration of observational and instructional learning. Simulation results for an example scenario can be found in Section 5. In Section 6, a detailed analysis of equilibria is addressed by which verification of the model was performed. Section 7 is for discussion.

## 2 Overview of background knowledge on mental models

Mental models are being studied in Cognitive and Social Sciences, as well as in Educational Sciences for a long time, e.g., [1–16, 27–35]. A famous quote of Craik from his 1943 book mentions:

‘If the organism carries a “small-scale model” of external reality and of its own possible actions within its head, it is able to try out various alternatives, conclude which is the best of them, react to future situations before they arise, utilise the knowledge of past events in dealing with the present and future, and in every way to react in a much fuller, safer, and more competent manner to the emergencies which face it.’

(Craik [2], p. 61)

He writes that such internal models use a certain *relation-structure* that makes the mental model work in a way similar to how the real world works:

‘By “relation-structure” I do not mean some obscure non-physical entity which attends the model, but the fact that it is a physical working model which works in the same way as the process it parallels…

(Craik [2], p. 51).

Within educational science, the term model-based learning is used for learning based on constructing coherent mental models [1, 9, 36–38]. Buckley formulates this as:

‘Model-based learning is a dynamic, recursive process of learning by building mental models.’

(Buckley [1])

More specifically, the following elements play an important role in such learning.

### 2.1 Learning by observation

Observational learning occurs when observation of others is the main source for the formation of a mental model. For example, the trainees see someone else perform a target behavior and then attempt to imitate or reenact it; e.g., [36, 39]. Demonstration is an often applied method to let others learn a specific motor task. This type is called observational motor learning. Empirical research has found that observational motor learning improves action perception and motor execution. From a neuroscientific perspective, mirror neurons are considered responsible for the ability to learn by observing and imitating others, e.g., [40–42].

### 2.2 Learning by instruction

Instructional learning assumes that instructions from an expert instructor are supportive for learning. For a beginner, only learning by discovery or observation often involves much trial and error; e.g., [14, 15]. Hence, instructions from an expert are considered a useful addition to build effective mental models. This is supported by a format of scaffolded model-based learning in which many supporting actions such as prompts, questions, hints, stories, conceptual models, visualizations are performed to facilitate a learner’s progress during learning tasks, e.g., [43].

### 2.3 Learner-controlled learning

For the integration of observational and instructional learning, control is a crucial element. It is discussed, for example, by Gibbons and Gray [44] that instructions serve learning processes best when the learner has control over them. The scaffolded model-based learning format mentioned above supports this. Kozma [28] suggested that individuals actively use external information sources for mental model formation. Learners are sensitive to characteristics of the learning environment such as the availability of certain information at a given time, the structure of the information and the ease with which it can be accessed. Thus, the learner’s need for instruction and the ease acquiring it are crucial for building effective mental models. In learning methods based on guided discovery, the learner seeks for information to complete the initial mental model. This requires the learner to be proactive and in control of the learning process. In contrast, in expository teaching methods, an instructor aims at directing the mental model formation by providing adequate information according to some temporal sequence [33]. Meela, and Yuenyong [29] demonstrated in their study that Model-Based Inquiry (MBI) can support a student’s mental model formation in scientific learning. MBI focuses on students’ formulations of questions and procedures [31]. Feedback on performance are a significant factor in learning [1, 45]; many studies support that feedback is crucial in skill acquisition [46].

Thus, in the adaptive network model introduced in the current paper, the learner can seek for instructions whenever it is useful or needed or as a feedback about what she/he has learnt by observation. The control for this was modeled by control states for instructions on a separate level within the adaptive network model. Using this, the learner controls timing and content of incoming information by seeking it only when it seems appropriate to her/him. More specifically, in Section 3 it is shown how a learning process based on mental models can be modeled by a generic three-level adaptive network architecture. In this architecture, the mental models themselves can be modeled at the base level as networks. In addition, during learning the mental models change; this can be modeled by (first-order) network adaptation at a second level. Control of the learning process is a form of adaptation of the learning process; this can be modeled at the third level addressing adaptation of the first-order adaptive network for the learning process: *second-order network adaptation*.

## 3 Network architecture for controlled mental model learning

In this section a global view on the architecture of the introduced network model for learner-controlled mental model learning is discussed. Following with what was concluded in Section 2, this architecture must cover the following three types of processes in an integrated manner:

1. The mental models themselves described by base networks
2. Learning as change of mental models described by first-order network adaptation
3. Control of learning processes described by second-order network adaptation.

**Fig 1:**
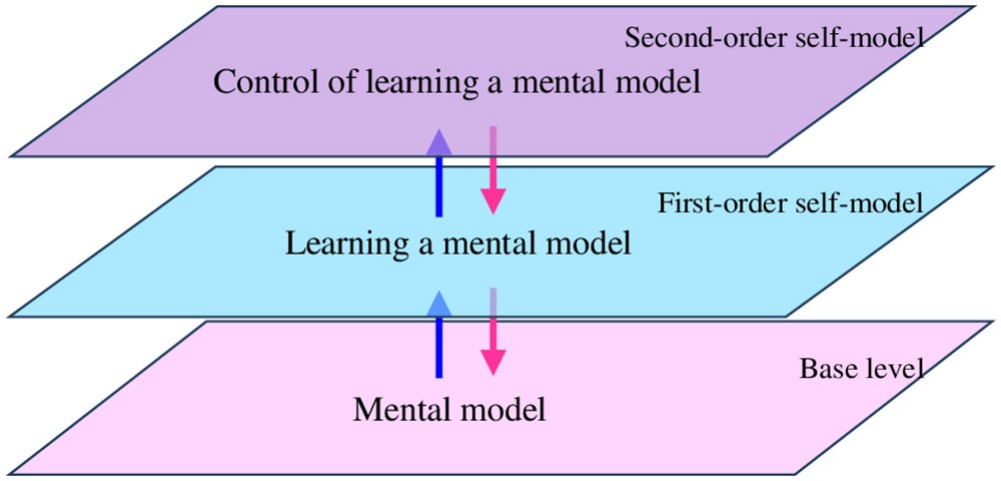
Overview of the introduced second-order adaptive network architecture.

Using the notion of self-modeling network (also called reified network) [22–24], these three description levels indeed can be modeled adequately by a three-level second-order adaptive network architecture as depicted in Fig 1.

Here, for any specific application each plane contains a specific network and the specific upward and downward connections define the interactions between the different levels.

More specifically, adding a self-model to a network model is done in the way that for some of the network structure characteristics additional network states (self-model states) are added. In the network-oriented modeling approach [23] applied here, in particular for nodes (also called states) *X* and *Y*, the following network structure characteristics are used:

- **ω**_*X*,*Y*_ for *connectivity* (connections *X* → *Y* with their connection weights)
- **γ**_*i*,*Y*_ and **π**_*i*,*j*,*Y*_ for *aggregation* (combination function choices and their parameters for each node *Y*)
- **η**_*Y*_ for *timing* (speed factors for each node *Y*)

Then to obtain adaptive networks, self-model nodes can be added to the network for any of these characteristics to make it adaptive:

- **Connectivity self-model**
  Self-model nodes **W**_*X*,*Y*_ are added representing connection weights **ω**_*X*,*Y*_
- **Aggregation self-model**
  Self-model nodes **C**_*j*,*Y*_ are added representing combination function weights **γ**_*i*,*Y*_ and/or self-model states **P**_*i*,*j*,*Y*_ representing combination function parameters **π**_*i*,*j*,*Y*_
- **Timing self-model**
  Self-model nodes **H**_*Y*_ are added representing speed factors **η**_*Y*_

The notations **W**_*X*,*Y*_, **C**_*i*,*Y*_, **P**_*i*,*j*,*Y*_, **H**_*Y*_ for the self-model states indicate the referencing relation with respect to the characteristics **ω**_*X*,*Y*_, **γ**_*i*,*Y*_, **π**_*i*,*j*,*Y*_, **η**_*Y*_: here **W** refers to **ω**, **C** refers to **γ**, **P** refers to **π**, and **H** refers to **η**, respectively. These **W**, **C**, **P** and **H** notations are considered to indicate the roles these **W**-, **C**-, **P**- and **H**-states play in the network, so that at the base level the values of them are used for the intended characteristics. Sometimes slightly different notations are used, for example, by adding the letter **R** for representation to emphasize that it represents some characteristic of the network: **RW**_*X*,*Y*_, **RC**_*i*,*Y*_, **RP**_*i*,*j*,*Y*_, **RH**_*Y*_. This construction can easily be applied iteratively to obtain multiple levels of self-models. For example, by adding a second-order self-model state **H**_**W***X*,*Y*_ for **W**_*X*,*Y*_, the adaptation speed **η**_**W***X*,*Y*_ of **W**_*X*,*Y*_ can be made adaptive. Therefore second-order adaptation plays an important role here to control adaptive processes which can be easily modelled as well.

The more specific adaptive network model described in Section 4 will be a refinement of the overall network architecture depicted in Fig 1. Tables 1 and 2 summaries the generic types of states and connections used at and between the three levels within this architecture. Note that the colours used in these tables indicate to which level the states belong, as they correspond to the colours of the planes in the 3D figures such as Fig 1.

**Table 1:** Types of states in the introduced three level network architecture.

BS_*Y*_	Base states for the considered mental model of the learner
OS_*Y*_	The corresponding observation states in the real world
**IS**_*X*,*Y*_	Representation for the connection weights of the mental model of the instructor
**LW**_*X*,*Y*_	Representation for the connection weights for the mental model as learnt from observation (using the Hebbian learning principle)
**IW**_*X*,*Y*_	Representation for the connection weights for the mental model as learnt from instruction (using the instructor)
**RW**_*X*,*Y*_	Representation for the connection weights for the learner’s mental model integrating observational (via **LW**_*X*,*Y*_) and instructional (via **IW**_*X*,*Y*_) learning
**CIW**_*X*,*Y*_	Initiation of instruction: control state for requesting the weight of the connection from *X* to *Y* for the mental model from the instructor

**Table 2:** Types of connections in the introduced adaptive network architecture.

**Intralevel connections**		
BS_*X*_ → BS_*Y*_	The learner’s (subjective) connections between the base states, indicating the current mental model of the learner	
OS_*X*_ → OS_*Y*_	The real world’s (objective) connections between the observation states, indicating the real-world process	
OS_*Y*_ → BS_*Y*_	Mirroring connections defining the mirroring process for the base states. These connections model the effect of observations on the learner.	
**IS**_*X*,*Y*_ → **IW**_*X*,*Y*_	Being informed by the instructor: the communicated instruction concerning the connection from *X* to *Y*. These connections **IS**_*X*,*Y*_ → **IW**_*X*,*Y*_ can be controlled by control states **CIW**_*X*,*Y*_ at the second reification level	
**IW**_*X*,*Y*_ → **RW**_*X*,*Y*_	Integration of knowledge obtained by instructional learning	
**LW**_*X*,*Y*_ → **RW**_*X*,*Y*_	Integration of knowledge obtained by observational learning	
**Interlevel connections**		
BS_*X*_ → **LW**_*X*,*Y*_ BS_*Y*_ → **LW**_*X*,*Y*_	Connections supporting observational learning	Upward from base level to first reification level
**RW**_*X*,*Y*_ → BS_*Y*_	Effectuation of base connection weights in the mental model	Downward from first reification level to base level
**LW**_*X*,*Y*_ → **CIW**_*X*,*Y*_	Observational learning monitoring connections	Upward from first to second reification level
**CIW**_*X*,*Y*_ → **IW**_*X*,*Y*_	Effectuation of instructional learning control	Downward from second to first reification level

**Fig 2:**
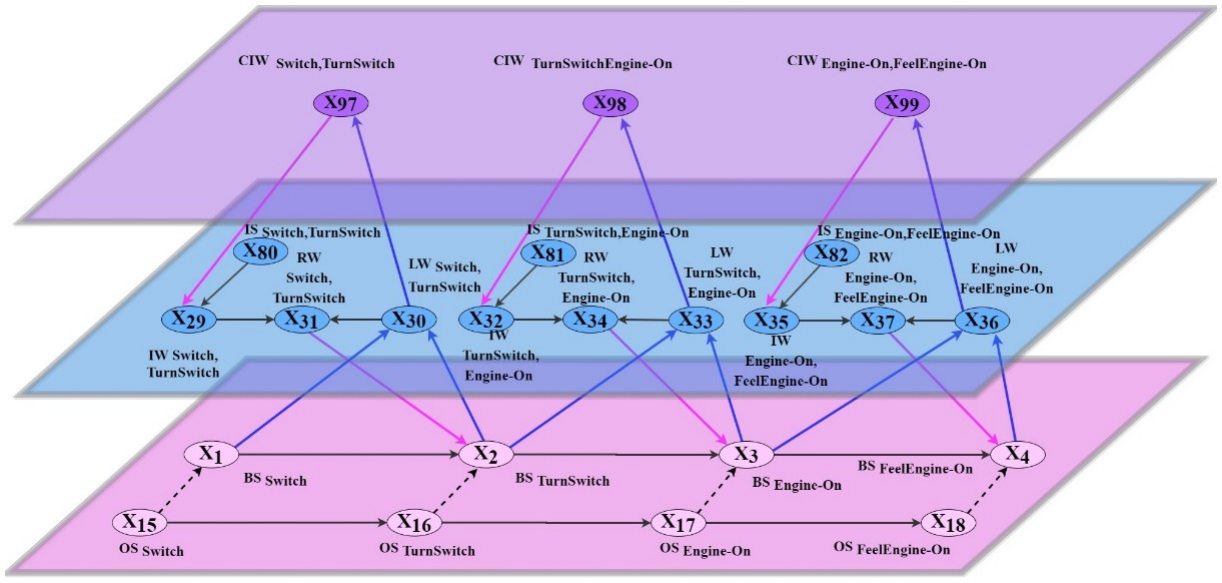
Connectivity for part of the second-order adaptive network model.

**Fig 3:**
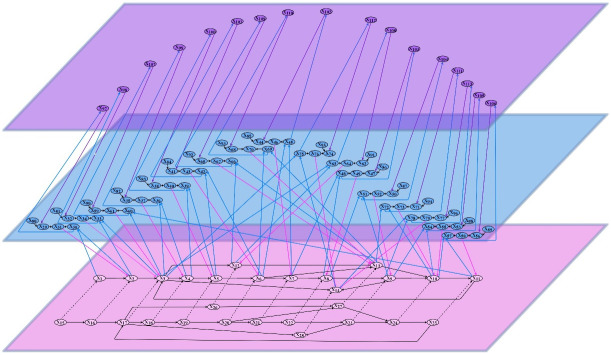
Connectivity for the complete adaptive network model.

At the base level, the learner’s (subjective) mental model is defined by connections between base states BS_*X*_ → BS_*Y*_; in addition, the connections between observation states OS_*X*_ → OS_*Y*_ define the (objective) relations in the real world. Note that, following the quote of Craik [6], p. 51 in Section 2, the causal relations BS_*X*_ → BS_*Y*_ defining the mental model, are in a one-to-one correspondence with the causal relations OS_*X*_ → OS_*Y*_ between the (observed) world states. Therefore, as can be seen in Figs 2 and 3, within the base plane the subnetwork for the BS-states has a connectivity structure that is isomorphic to the connectivity structure of the subnetwork for the OS-states.

Moreover, the connections from observation state to base state OS_*Y*_ → BS_*Y*_ define the mirroring process by which the observations affect the learner’s own states.

At the first-order self-model level, the self-model of the mental model from the base level is modeled by states **RW**_*X*,*Y*_ that explicitly represent the connection weights of the mental model as used in the processing of this mental model at the base level. At first sight, this may seem a double (and therefore redundant) representation of the same mental model, but to handle the process of learning of this mental model well, this explicit ‘additional’ representation in the form of the mental model’s self-model is crucial, because when adaptivity is addressed, the network characteristic (in this case connection weight) is no longer one static parameter value but becomes a variable with values that change over time (as happens for every dynamical system model for which some of its parameters are made adaptive). The way of conceptualization applied here is used more often within neuroscience as a distinction between (1) activation propagation through a network of neurons, (2) plasticity of this network, and (3) metaplasticity as control over this plasticity; e.g., [25, 47–50]. Inspired by this, in the current paper, (1) and (2) of this form of conceptualization are used to model use and adaptation of a mental model, respectively, and (3) for the control over this adaptation, as will be explained in more detail below.

In addition to the states **RW**_*X*,*Y*_, also self-model states **LW**_*X*,*Y*_ and **IW**_*X*,*Y*_ are used as part of the mental model’s self-model. Here, **LW**_*X*,*Y*_ represents what has been learnt about the connection from *X* to *Y* within the mental model by observational learning and **IW**_*X*,*Y*_ represents what has been acquired from instructional learning.

The intra-level connections **LW**_*X*,*Y*_ → **RW**_*X*,*Y*_ and **IW**_*X*,*Y*_ → **RW**_*X*,*Y*_ model the integration within **RW**_*X*,*Y*_ of what is learnt by observational learning and what is learnt by instructional learning. The connections **IS**_*X*,*Y*_ → **IW**_*X*,*Y*_ model the instruction itself: the communication actions from instructor to learner.

These communication actions from the instructor to the learner depend on control. To this end, in the second-order self-model, states **CIW**_*X*,*Y*_ are included. Such a state indicates that the learner wants to hear the instructor’s knowledge about the connection from *X* to *Y*. It is assumed that the instructor will respond accordingly. This happens by giving **CIW**_*X*,*Y*_ the role of connection weight representation **W**_**IS***X*,*Y*,**IW***X*,*Y*_ for the intended connection **IS**_*X*,*Y*_ → **IW**_*X*,*Y*_ from the instructor to the learner. This works by the processing of the first-order self-model for the connection weight **ω**_**IS***X*,*Y*,**IW***X*,*Y*_ the value of **CIW**_*X*,*Y*_ is used. Therefore, as long as the value of **CIW**_*X*,*Y*_ is 0, no communication takes place, while as soon as this value of **CIW**_*X*,*Y*_ is 1, this communication does take place. This represents the way in which that communication becomes controlled. The effect of activation of **CIW**_*X*,*Y*_ can be interpreted in the sense that the communication channel from the instructor state **IS**_*X*,*Y*_ to the learner state **IW**_*X*,*Y*_ is opened, so that this information is transferred from the instructor state **IS**_*X*,*Y*_ to the learner state **IW**_*X*,*Y*_. This opening of the channel **IS**_*X*,*Y*_ → **IW**_*X*,*Y*_ is modeled by the downward connections **CIW**_*X*,*Y*_ → **IW**_*X*,*Y*_, where **CIW**_*X*,*Y*_ represents the role of connection weight from **IS**_*X*,*Y*_ to **IW**_*X*,*Y*_.

The only remaining piece then is to determine when exactly **CIW**_*X*,*Y*_ should become active. This is done via its incoming observational learning monitoring connection **LW**_*X*,*Y*_ → **CIW**_*X*,*Y*_ which makes that the control state **CIW**_*X*,*Y*_ will become active depending on the corresponding **LW**-state **LW**_*X*,*Y*_. This models that the part where the learner asks the instructor for verification and confirmation of what was just learnt by observation (and the learner does not ask anything about what not yet has been observed). A more detailed explanation of the network’s connectivity for a specific case study can be found in Section 4.

## 4 Detailed description of the second-order adaptive network model for a case study

In this section, a more detailed description can be found of the designed second-order adaptive network model for a realistic case study, that was created for illustrative purposes. It is described by the following scenario:

Person A has almost no knowledge about a car’s components and their interplay and how to drive a car. This person’s mental model of the car and driving it has to be learned during driving lessons. During person A’s first driving lesson, instructor B demonstrates how to start a car and get it moving. The observation of B makes that A learns an initial mental model of the car and how it can be operated it (observational learning). During A’s further learning, an iterative process of extending and/or modifying the mental model takes place, leading to a more accurate and complete mental model. Besides observational learning, also learning from instruction plays an important role (instructional learning). This instructional learning takes place by incorporating incoming information communicated by B. In this scenario this instructional learning only takes place upon request of the learner (learner-controlled instructional learning), as a form of verification and consolidation after A learnt about it by observational learning.

The network-oriented modeling approach for adaptive networks [22–24] used here has been briefly introduced in Section 3. Some more details will follow here. Recall that for adaptive networks the notion of *self-modeling network* is used. For example, for adaptive connectivity characteristics, states **RW**_*X*,*Y*_ are added representing adaptive connection weights **ω**_*X*,*Y*_. They form a *self-model* of the network’s own structure in the form of a subnetwork within the network. To graphically distinguish them from states at the level of *X* and *Y*, these self-model states are depicted at one level higher (e.g., see the blue planes in Figs 1–3 with representations of weights of adaptive connections from the base planes).

As in this case the learning is controlled, it is adaptive itself, which is depicted by the third level (purple plane) for *second-order adaptation* in Figs 1–3, which include *second-order reification states* **CIW**_*X*,*Y*_ that represent the weight of the connection **IS**_*X*,*Y*_ → **IW**_*X*,*Y*_ of the middle level (see Section 3). The structure formed by the lowest two (interacting) levels distinguish the two types of processes (and their interaction): *using* the mental model by changing the **BS**-states represented at the base level (used for *internal simulation* of the mental model) versus *adjusting* the mental model by changing the representations at the self-model level. The different types of states for the detailed model are explained in Tables 4, 5, 6. Fig 2 depicts the connectivity for only a part for a small number of the states for better understanding. Fig 3 shows the connectivity for the complete network model. The second-order self-model level (the purple plane) enables to control the learning process by changing some of the intra-level connections within the first-order self-model (which in turn affects the dynamics of these first-order self-model states), based on the second-level reification **CIW**-states (control states); this is used to model *learner-controlled instruction*, as discussed in Section 3.

The conceptual representation of a network model as mentioned above can easily be transformed in an automated manner into a numerical representation using a dedicated modeling environment; within the software, this results in difference equations ([22, 23], Chapter 9):

$$\begin{array}{ll} & Y(t+\Delta t)=Y(t)+\eta_{Y}\left[{\text{aggimpact}}_{Y}(t)-Y(t)\right]\;\Delta t\\ \text{or} & \text{d}Y\left(t\right)/\text{d}t=\eta_{Y}\left[{\text{aggimpact}}_{Y}(t)-Y(t)\right]\\ \text{where} & {\text{aggimpact}}_{Y}(t)=c_{Y}(\omega_{X_{1},Y}X_{1}\left(t\right),\ldots ,\omega_{X_{{}_{k}},Y}X_{k}\left(t\right)) \end{array}$$ (1)

Here the overall combination function **c**_*Y*_**(‥)** for state *Y* is the weighted average of available basic combination functions **c**_*j*_**(‥)** (in the Combination Function Library) by specified weights **γ**_*j*,*Y*_ and parameters **π**_1,*j*,*Y*_, **π**_2,*j*,*Y*_ of **c**_*j*_**(‥)** for *Y*:

$$c_{Y}(V_{1},\ldots ,V_{k})=\frac{\gamma_{1,Y}{\text{c}}_{1}\left(V_{1},\;\ldots ,\;V_{k}\right)+\ldots +\gamma_{m,Y}{\text{c}}_{m}\left(V_{1},\;\ldots ,\;V_{k}\right)}{\gamma_{1,Y}+\ldots +\gamma_{m,Y}}$$ (2)

In case of self-models, the self-model states define the dynamics of state *Y* in a canonical manner according to (1), whereby the adaptive characteristics among **ω**_*X*,*Y*_, **γ**_*i*,*Y*_, **π**_*i*,*j*,*Y*_, **η**_*Y*_ are replaced by the state values of self-model states **W**_*X*,*Y*_, **C**_*i*,*Y*_, **P**_*i*,*j*,*Y*_, **H**_*Y*_ at time *t*, respectively (for more details, see [22, 23]).

In the model presented here, for the states the following combination functions were used, all generating values in [0, 1] (assuming that their arguments are in [0, 1]). The *Euclidean combination function* **eucl**_**n,λ**_**(***V*_1_, *…*, *V*_*k*_**)** where ***n*** is the order (any positive number), and **λ** the scaling factor is defined by:

$${\text{eucl}}_{n,\lambda }(V_{1},\ldots ,V_{k})=\sqrt[{n}]{\frac{V_{1}^{n}+\cdots +V_{k}^{n}}{\lambda }}$$ (3)

where *V*_1_, *…*, *V*_*k*_ ∈ [0, 1] indicate the impacts **ω**_*Xi*,*Y*_ *X*_*i*_ *(t)* from the states *X*_1_, *…*, *X*_*k*_ from which *Y* has an incoming connection. In addition, the *advanced logistic sum combination function* **alogistic**_**σ,τ**_**(…)** is used:

$${\text{alogistic}}_{\sigma ,\tau }(V_{1},\ldots ,V_{k})=\left[\frac{1}{1+{\text{e}}^{-\sigma }\left(V_{1}+\cdots +V_{k}-\tau \right)}-\frac{1}{1+{\text{e}}^{\sigma \tau }{}^{)}}\right]\left(1+{\text{e}}^{-\sigma \tau }\right)$$ (4)

with steepness **σ** and threshold **τ** (with similar *V*_1_, *…*, *V*_*k*_ as above)

Table 3 provides an overview of the base states used to model the mental model (the BS-states) and the base states for the observations (the OS-states). Table 4 summarises the first-order self-model states for the learner’s learning (the **RW**-, **LW**- and **IW**-states) and Table 5 addresses the instructor’s Information States (the **IS**-states).

**Table 3:** Explanation of the base level states for the mental model within the network model.

Base States		Explanation
X_1_	BS_Switch_	Learner’s representation state for Switch
X_2_	BS_TurnSwitch_	Learner’s representation state for TurnSwitch
X_3_	BS_Engine-0n_	Learner’s representation state for Engine-On
X_4_	BS_FeelEngine-On_	Learner’s representation state for FeelEngine-On
X_5_	BS_PresClutch_	Learner’s representation state for PressClutch
X_6_	BS_Clutch-On_	Learner’s representation state for Clutch-On
X_7_	BS_Gearbox-Neutral_	Learner’s representation state for Gearbox-Neutral
X_8_	BS_PressGear 1_	Learner’s representation state for PressGear1
X_9_	BS_Gear1-On_	Learner’s representation state for Gear1-On
X_10_	BS_PressAccelerator_	Learner’s representation state for PressAccelerator
X_11_	BS_Accelerator-On_	Learner’s representation state for Accelerator-On
X_12_	BS_RevMeter-On_	Learner’s representation state for Rev-Meter-On
X_13_	BSe_BiteState_	Learner’s representation state for BiteState
X_14_	BS_MovingState_	Learner’s representation state for MovingState
X_15_	OS_Switch_	Observation State for Switch
X_16_	OS_TurnSwitch_	Observation State for TurnSwitch
X_17_	OS_Engine-0n_	Observation State for Engine-On
X_18_	OS_FeelEngine-On_	Observation State for FeelEngine-On
X_19_	OS_PressClutch_	Observation State for PressClutch
X_20_	OS_Clutch-On_	Observation State for Clutch-On
X_21_	OS_Gearbox-Neutral_	Observation State for Gearbox-Neutral
X_22_	OS_PressGear 1_	Observation State for PressGear1
X_23_	OS_Gear1_	Observation State for Gear1-On
X_24_	OS_PressAccelerator_	Observation State for PressAccelerator
X_25_	OS_Accelerator-On_	Observation State for Accelerator-On
X_26_	OS_RevMeter-On_	Observation State for Rev-Meter-On
X_27_	OS_BiteState_	Observation State for BiteState
X_28_	OS_MovingState_	Observation State for MovingState

**Table 4:** Explanation of the first-order self-model states for the mental model learning in the network model.

First-order self-model states for the learning		Explanation
X_29_	**IW**_Switch,TurnSwitch_	Representation state for Informed Connection Weight Switch→TurnSwitch
X_30_	**LW**_Switch,TurnSwitch_	Representation state for Learnt Connection Weight Switch→TurnSwitch
X_31_	**RW**_Switch,TurnSwitch_	Representation state for overall Connection Weight Switch→TurnSwitch
X_32_	**IW**_TurnSwitch,Engine-On_	Representation state for Informed Connection Weight TurnSwitch→Engine-On
X_33_	**LW**_TurnSwitch,Engine-On_	Representation state for Learnt Connection Weight TurnSwitch→Engine-On
X_34_	**RW**_TurnSwitch,Engine-On_	Representation state for overall Connection Weight TurnSwitch→Engine-On
X_35_	**IW**_Engine-On,FeelEngine-On_	Representation state for Informed Connection Weight Engine-On→FeelEngine-On
X_36_	**LW**_Engine-On,FeelEngine-On_	Representation state for Learnt Connection Weight Engine-On→FeelEngine-On
X_37_	**RW**_Engine-On,FeelEngine-On_	Representation state for overall Connection Weight Engine-On→FeelEngine-On
X_38_	**IW**_FeelEngine-On,PressClutch_	Representation state for Informed Connection Weight FeelEngine-On→PressClutch
X_39_	**LW**_eelEngine-On,PressClutch_	Representation state for Learnt Connection Weight FeelEngine-On→PressClutch
X_40_	**RW**_FeelEngine-On,PressClutch_	Representation state for overall Connection Weight FeelEngine-On→PressClutch
X_41_	**IW**_PressClutch,Clutch-On_	Representation state for Informed Connection Weight PressClutch→Clutch-On
X_42_	**LW**_PressClutch,Clutch-On_	Representation state for Learnt Connection Weight PressClutch→Clutch-On
X_43_	**RW**_PressClutch,Clutch-On_	Representation state for overall Connection Weight PressClutch→Clutch-On
X_44_	**IW**_Clutch-On,Gearbox-Neutral_	Representation state for Informed Connection Weight Clutch-On→Gearbox-Neutral
X_45_	**LW**_Clutch-On,Gearbox-Neutral_	Representation state for Learnt Connection Weight Clutch-On→Gearbox-Neutral
X_46_	**RW**_Clutch-On,Gearbox-Neutral_	Representation state for overall Connection Weight Clutch-On→Gearbox-Neutral
X_47_	**IW**_Gearbox-Neutral,PressGear1_	Representation state for Informed Connection Weight Gearbox-Neutral→PressGear1
X_48_	**LW**_Gearbox-Neutral,PressGear1_	Representation state for Learnt Connection Weight Gearbox-Neutral→PressGear1
X_49_	**RW**_Gearbox-Neutral,PressGear1_	Representation state for overall Connection Weight Gearbox-Neutral→PressGear1
X_50_	**IW**_PressGear1,Gear1-On_	Representation state for Informed Connection Weight PressGear1→Gear1-On
X_51_	**LW**_PressGear1,Gear1-On_	Representation state for Learnt Connection Weight PressGear1→Gear1-On
X_52_	**RW**_PressGear1,Gear1-On_	Representation state for overall Connection Weight PressGear1→Gear1-On
X_53_	**IW**_Gear1-On,PressAccelerator_	Representation state for Informed Connection Weight Gear1-On→PressAccelerator
X_54_	**LW**_Gear1-On,PressAccelerator_	Representation state for Learnt Connection Weight Gear1-On→PressAccelerator
X_55_	**RW**_Gear1-On,PressAccelerator_	Representation state for overall Connection Weight Gear1→On,PressAccelerator
X_56_	**IW**_PressAccelerator,Accelerator-On_	Representation state for Informed Connection Weight PressAccelerator→Accelerator-On
X_57_	**LW**_PressAccelerator,Accelerator-On_	Representation state for Learnt Connection Weight PressAccelerator→Accelerator-On
X_58_	**RW**_PressAccelerator,Accelerator-On_	Representation state for Connection Weight PressAccelerator→Accelerator-On
X_59_	**IW**_Accelerator-On,Engine-On_	Representation state for Informed Connection Weight Accelerator-On→Engine-On
X_60_	**LW**_Accelerator-On,Engine-On_	Representation state for Learnt Connection Weight Accelerator-On→Engine-On
X_61_	**RW**_Accelerator-On,Engine-On_	Representation state for overall Connection Weight Accelerator-On→Engine-On
X_62_	**IW**_Engine-On,Rev-Meter-On_	Representation state for Informed Connection Weight Engine-On→Rev-Meter-On
X_63_	**LW**_Engine-On,Rev-Meter-On_	Representation state for Learnt Connection Weight Engine-On→Rev-Meter-On
X_64_	**RW**_Engine-On,Rev-Meter-On_	Representation state for overall Connection Weight Engine-On→Rev-Meter-On
X_65_	**IW**_RevMeter-On,BiteState_	Representation state for Informed Connection Weight RevMeter-On→BiteState
X_66_	**LW**_RevMeter-On,BiteState_	Representation state for Learnt Connection Weight RevMeter-On→BiteState
X_67_	**RW**_RevMeter-On,BiteState_	Representation state for overall Connection Weight RevMeter-On→BiteState
X_68_	**IW**_Clutch-On,BiteState_	Representation state for Informed Connection Weight Clutch-On→BiteState
X_69_	**LW**_Clutch-On,BiteState_	Representation state for Learnt Connection Weight Clutch-On→BiteState
X_70_	**RW**_Clutch-On,BiteState_	Representation state for overall Connection Weight Clutch-On→BiteState
X_71_	**IW**_BiteState,PressAccelerator_	Representation state for Informed Connection Weight BiteState→PressAccelerator
X_72_	**LW**_BiteState,PressAccelerator_	Representation state for Learnt Connection Weight BiteState→PressAccelerator
X_73_	**RW**_BiteState,PressAccelerator_	Representation state for overall Connection Weight BiteState→PressAccelerator
X_74_	**IW**_Engine-On,MovingState_	Representation state for Informed Connection Weight Engine-On→MovingState
X_75_	**LW**_Engine-On,MovingState_	Representation state for Learnt Connection Weight Engine-On→MovingState
X_76_	**RW**_Engine-On,MovingState_	Representation state for overall Connection Weight Engine-On→MovingState
X_77_	**IW**_Gear1-On,MovingState_	Representation state for Informed Connection Weight Gear1-On→MovingState
X_78_	**LW**_Gear1-On,MovingState_	Representation state for Learnt Connection Weight Gear1-On→MovingState
X_79_	**RW**_Gear1-On,MovingState_	Representation state for overall Connection Weight Gear1-On→MovingState

**Table 5:** Explanation of the first-order self-model states for the instructor’s Information States in the network model.

First-order self-model states for the instructor’s information		Explanation
X_80_	IS_Switch,TurnSwitch_	Representation state for Information State for Switch→TurnSwitch
X_81_	IS_TurnSwitch,Engine-On_	Representation state for instructor’s Information State for TurnSwitch→Engine-On
X_82_	IS_Engine-On,FeelEngine-On_	Representation state for instructor’s Information State for Engine-On→FeelEngine-On
X_83_	IS_FeelEngineOn,PressClutch_	Representation state for instructor’s Information State for FeelEngineOn→PressClutch
X_84_	IS_PressClutch,Clutch-On_	Representation state for instructor’s Information State for PressClutch→Clutch-On
X_85_	IS_Clutch-On,Gear-BoxNeutral_	Representation state for instructor’s Information State for Clutch-On→Gear-BoxNeutral
X_86_	IS_GearBox-Neutral,PressGear1_	Representation state for instructor’s Information State for GearBox-Neutral,PressGear1
X_87_	IS_PressGear1,Gear1-On_	Representation state for instructor’s Information State for PressGear1→Gear1-On
X_88_	IS_Gear1-On, PressAccelerator_	Representation state for instructor’s Information State for Gear1-On→PressAccelerator
X_89_	IS_PressAccelerator, Accelerator-On_	Representation state for instructor’s Information State for PressAccelerator→Accelerator-On
X_90_	IS_Accelerator-On, Engine-On_	Representation state for instructor’s Information State for Accelerator-On→Engine-On
X_91_	IS_Engine-On,Rev-Meter-On_	Representation state for instructor’s Information State for Engine-On→Rev-Meter-On
X_92_	IS_RevMeter-On,BiteState_	Representation state for instructor’s Information State for RevMeter-On→BiteState
X_93_	IS_Clutch-On,BiteState_	Representation state for instructor’s Information State for Clutch-On→BiteState
X_94_	IS_BiteState,PressAccelerator_	Representation state for instructor’s Information State for BiteState→PressAccelerator
X_95_	IS_Engine-On,MovingState_	Representation state for instructor’s Information State for Engine-On→MovingState
X_96_	IS_Gear-On1,MovingState_	Representation state for instructor’s Information State for Gear-On1→MovingState

The *Hebbian learning combination function* **hebb**_**μ**_**(‥)** for learning of the connection from state *X* to state *Y*, and used in particular for the **LW**-states is defined by

$${\text{hebb}}_{\mu }(V_{1},V_{2},W)=V_{1}V_{2}\left(1-W\right)+\mu W$$ (5)

where **μ** is the persistence parameter, *V*_1_ stands for state value *X(t)*, *V*_2_ for *Y(t)*, and *W* for the learnt connection weight reification state value **LW**_*X*,*Y*_(*t*), which all are in the [0, 1] interval. Hebbian learning is a well-known adaptation principle addressing adaptive connectivity, which can be explained by:

‘When an axon of cell A is near enough to excite B and repeatedly or persistently takes part in firing it, some growth process or metabolic change takes place in one or both cells such that A’s efficiency, as one of the cells firing B, is increased.’

[25], p. 62

This is sometimes simplified (neglecting the phrase ‘one of the cells firing B’) to:

‘What fires together, wires together’

[51, 52]

In formula (5), the condition ‘what fires together’ is modeled by the part with the product *V*_1_*V*_2_, as that is high if both states *X* and *Y* have a high value and low otherwise. The factor (1*-W*) provides a kind of normalisation that makes that the value for *W* = **LW**_*X*,*Y*_(*t*) does not exceed 1. The term **μ***W* in (5) models persistency, where **μ** indicates the fraction of the previously learnt value *W* that persists per time unit; for example if **μ** = 0.9, then every time unit 10% is lost (also called extinction).

In Table 6 an overview can be found of the second-order self-model states. They all are **CIW**-states for the control of the instructional learning of certain connection weights. For these **CIW**-states the logistic sum combination function **alogistic**_**σ,τ**_**(***V*_1_, …,*V*_*k*_**)** is used.

**Table 6:** Explanation of the second-order self-model states for control of the mental model learning in the network model.

Second-order self-model states for the control of the learning		Explanation
X_97_	**CIW**_Switch,TurnSwitch_	Representation state for Control of Information Weight for Switch→TurnSwitch
X_98_	**CIW**_TurnSwitch,Engine-On_	Representation state for Control of Information Weight for TurnSwitch→EngineOn
X_99_	**CIW**_Engine-On,FeelEngine-On_	Representation state for Control of Information Weight for Engine-On→FeelEngineOn
X_100_	**CIW**_FeelEngineOn,PressClutch_	Representation state for Control of Information Weight for FeelEngineOn→PressClutch
X_101_	**CIW**_PressClutch,Clutch-On_	Representation state for Control of Information Weight for PressClutch→Clutch-On
X_102_	**CIW**_Clutch-On,Gear-BoxNeutral_	Representation state for Control of Information Weight for Clutch-On→Gear-BoxNeutral
X_103_	**CIW**_GearBox-Neutral,PressGear1_	Representation state for Control of Information Weight for GearBox-Neutral,PressGear1
X_104_	**CIW**_PressGear1,Gear1-On_	Representation state for Control of Information Weight for PressGear1→Gear1-On
X_105_	**CIW**_Gear1-On, PressAccelerator_	Representation state for Control of Information Weight for Gear1-On→PressAccelerator
X_106_	**CIW**_PressAccelerator, Accelerator-On_	Representation state for Control of Information Weight for PressAccelerator→Accelerator-On
X_107_	**CIW**_Accelerator-On, Engine-On_	Representation state for Control of Information Weight for Accelerator-On→Engine-On
X_108_	**CIW**_Engine-On,Rev-Meter-On_	Representation state for Control of Information Weight for Engine-On→Rev-Meter-On
X_109_	**CIW**_RevMeter-On,BiteState_	Representation state for Control of Information Weight for RevMeter-On→BiteState
X_110_	**CIW**_Clutch-On,BiteState_	Representation state for Control of Information Weight for Clutch-On→BiteState
X_111_	**CIW**_BiteState,PressAccelerator_	Representation state for Control of Information Weight for BiteState→PressAccelerator
X_112_	**CIW**_Engine-On,MovingState_	Representation state for Control of Information Weight for Engine-On→MovingState
X_113_	**CIW**_Gear-On1,MovingState_	Representation state for Control of Information Weight for Gear-On1→MovingState

## 5 Simulation results for an example scenario

**Fig 4:**
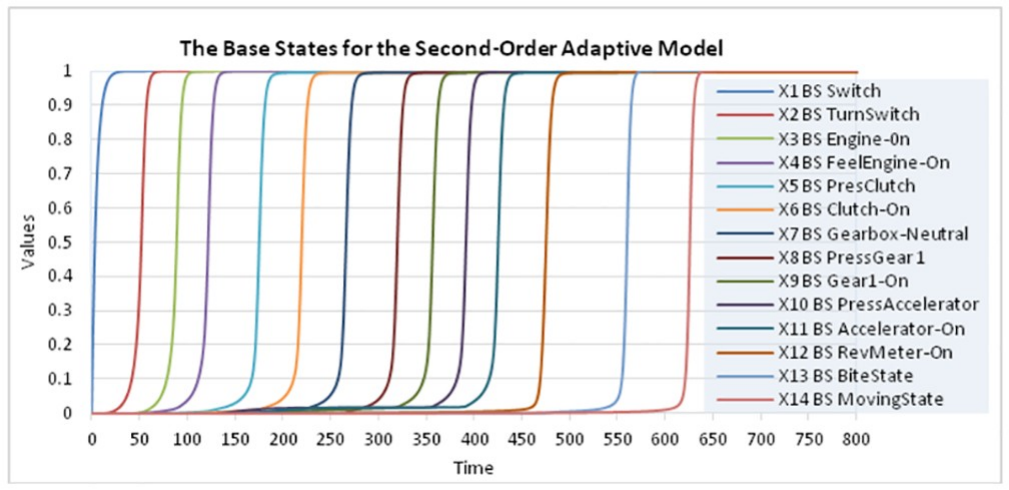
Dynamics of the base states X_1_-X_14_ showing internal simulation of the mental model.

The second-order adaptive network model was simulated using the dedicated software environment implemented in Matlab as described in [23], Ch 9, to study the learning of a mental model for a car’s functioning and driving it; see Fig 4 and further. For the simulation Δ*t* = 0.5 was chosen, the total time is 800 (so 1600 simulation steps); the time scale is left abstract here. In the S1 Appendix section the full specification of the network characteristics can be found. The speed factors for the BS-states were set at 0.4, for OS-states at 0.05, for **IW**-states at 0.1, and for **LW**-states and **RW**-states at 0.4. For the second-order **CIW**-states, their speed factors were set at 0.4. All BS- and OS-states use either the Euclidean function (3) or the logistic sum function (4), all **LW**-states the Hebbian learning function (5), the **IW**-states the logistic sum function (4), the **RW**-states the first-order Euclidean function (3), and the **CIW**-states the logistic sum function (4). All BS-states have initial value 0. All OS-States have initial value 0, except the first OS-State X_15_ which has an initial value of 1. For all the **IW**-, **LW**-, and **RW**- states, the initial value was set at 0.1.

**Fig 5:**
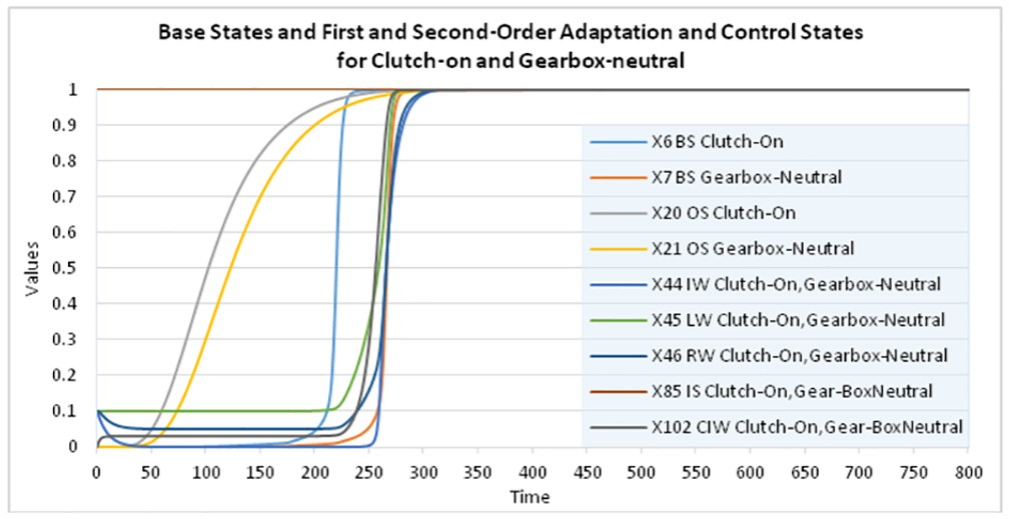
Base states X_6_ (Clutch-on) and X_7_ (Gearbox-neutral) with impact from OS-states X_20_, X_21_ and LW-state X_45_, RW-state X_46_ and learner IW-state X_44_ and instructor IS-state X_85_ with control by CIW-state X_102_.

**Fig 6:**
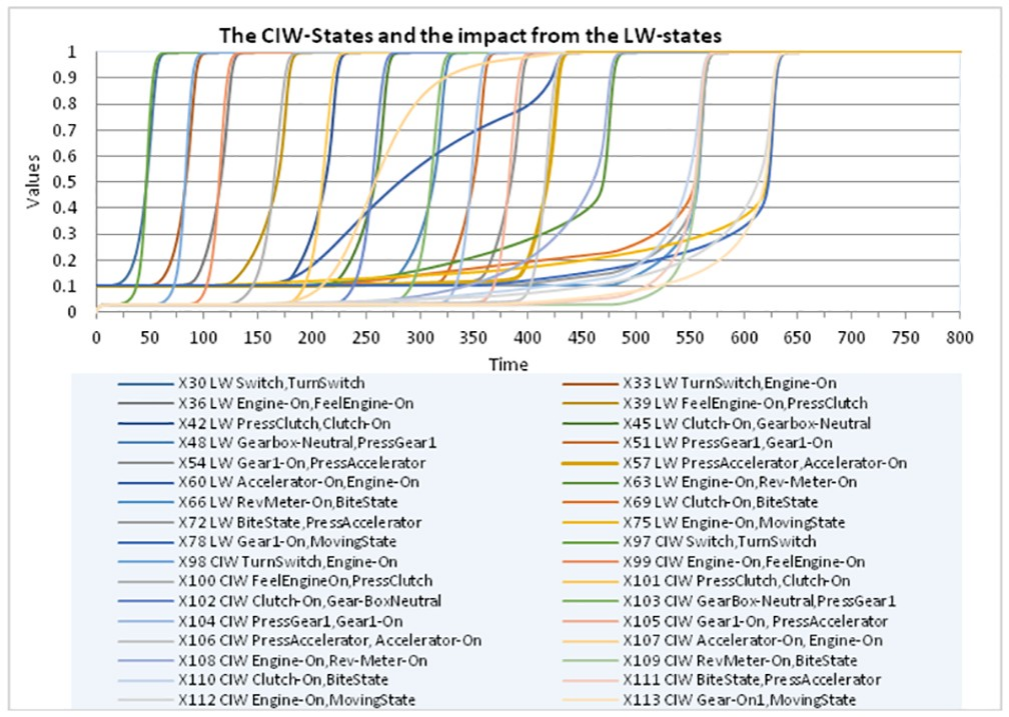
All control CIW-states showing impact from the corresponding observational learning LW-states.

**Fig 7:**
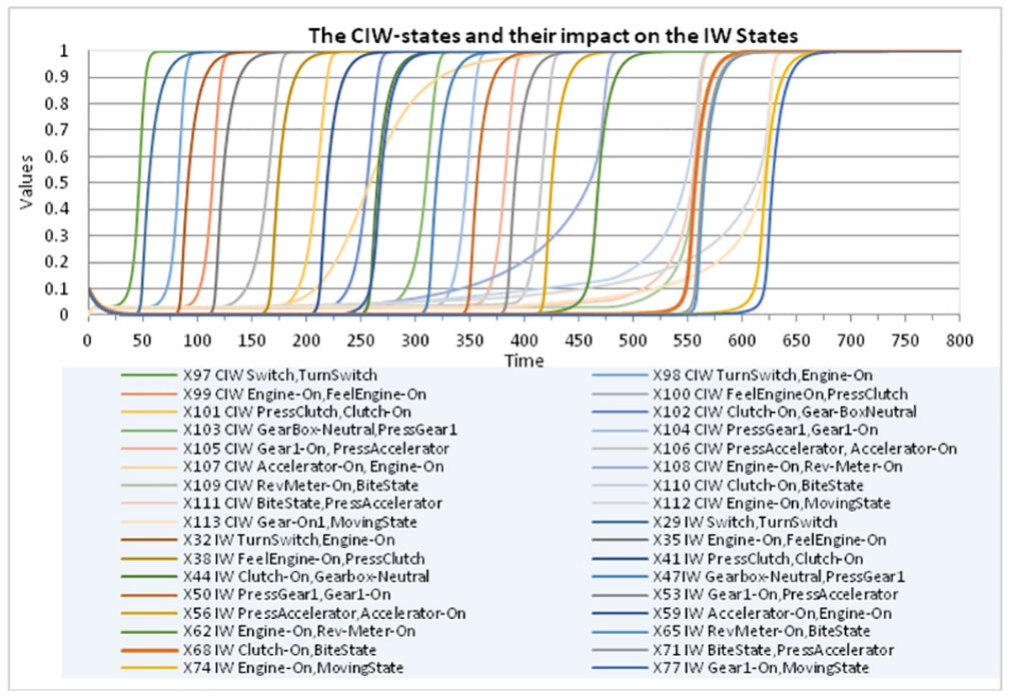
All control CIW-states showing their impact on the corresponding instructional learning IW-states.

The **IS**-states have constant value 1, as they refer to the knowledge of the instructor (see also Fig 5). Note that it has been specified in such a way that only one of the **IW**-state or **LW**-state is not enough to get a related **RW**-state with a high value close to 1. A typical pattern is that first, based on a learnt **LW**-state only, the **RW**-state gets a value somewhere in the middle of the 0–1 interval, and only after instructional learning making the **IW**-state high, the **RW**-state value increases to a high value close to 1. This makes that first based on the value of **LW**-state (i.e., by observational learning), the second-order **CIW**-state is activated (Fig 6) which in turn makes the **IW**-state getting a value close to 1 (Fig 7). Only after the learner seeks instructional information making the **IW**-state high, the **RW**-state value increases to 1. This shows that the learner actively engages seeking more information to confirm the accuracy of what he/she has learnt by observation.

The learner hence controls the amount of information (s)he needs in addition to complete her/his learning based on her/his current level of understanding by own observation (see also Section 3). The results indicated in Fig 5 display the connection between two **BS**-states X_6_ and X_7_. Here it can be seen that, together with the value of X_6_ becoming 1 at time 210, the **OS**-state X_21_ affects the value of X_7_ together with **RW**-state X_46,_ which combines the weights of the related **LW**-and **IW**-states. The **CIW**-state controls the weight of **IW**-state according to the **LW**-state’s weight. State X_7_ reaches value 1 at time 250 by an S-curve. The **IS**-state representing knowledge of the instructor remains at 1 all the time (a knowledgeable instructor).

As a form of evaluation, in Figs 6 and 7 it is displayed how the activation of each **CIW**-state indeed follows the activation of the corresponding **LW**-state, and how in turn the activation of the **CIW**-state indeed is followed by the corresponding **IW**-state. This confirms that the model displays the intended behavior that first observational learning takes place, after which there is a learner initiative to request corresponding instructional information, and appropriate instructional learning indeed takes place after that.

The simulation results presented by these figures are in accordance with and illustrate the educational science literature such as [28, 44] (as discussed in Section 2) on the use of learner-control of the timing of instruction.

## 6 Verification of the network model by equilibrium analysis

To verify whether the introduced and implemented self-modeling network model behaves as expected from its design specification, a number of network states equilibrium values were analyzed for the example simulation.

### 6.1 Criterion for equilibria of self-modeling network models

A *stationary point* for a state *Y* occurs at time *t* if d*Y(t)*/d*t* = 0. An *equilibrium* occurs when all states have a stationary point simultaneously. From Eq (1) in Section 4, for any state *Y* for being stationary the following general *criterion* in terms of the network characteristics can be derived:

$$\eta_{Y}=0\;\text{or}\;{\text{aggimpact}}_{Y}\left(t\right)=Y\left(t\right)$$ (6)

where

$${\text{aggimpact}}_{Y}\left(t\right)=c_{Y}(\omega_{X_{1},Y}X_{1}\left(t\right),\ldots ,\omega_{X_{k},Y}X_{k}\left(t\right))$$

with *X*_1_ to *X*_*k*_ the states from which *Y* gets its incoming connections.

The equation in (6) is also called an *equilibrium equation*. As a test, using the example simulation presented above for the apparent equilibrium at time 800, for 68 of the 113 network states it has been verified (independent of the implemented model) that the aggregated impact **aggimpact**_*Xi*_ **(***t***)** matches the state value for equilibrium values observed in the example simulation. In particular, this has been done for all 4x17 = 68 **LW**-, **IW-**, **RW**-, and **CIW**-states. The results are discussed in Sections 6.2 to 6.4.

### 6.2 Equilibrium analysis of the LW-states and the CIW-states

The **LW**-states use the Hebbian learning function **hebb**_μ_ **(***V*_1_, *V*_2_, *W***)** as combination function. Using this function, by (5) for any **LW**-state *Y* holds

$${\text{aggimpact}}_{Y}\left(t\right)={\text{hebb}}_{\mu }(V_{X_{1}},V_{X_{2}},V_{LW})=V_{X_{1}}V_{X_{2}}\left(1-V_{LW}\right)+\mu V_{LW}$$ (7)

where *V*_*X*1_, *V*_*X*2_ are the state values of the connected base states *X*_1_ and *X*_2_ and *V*_**LW**_ the state value of **LW**-state *Y*. So, for this case the equilibrium equation in (6) becomes

$$V_{X_{1}}V_{X_{2}}\left(1-V_{LW}\right)+\mu \;V_{LW}=V_{LW}$$ (8)

Assuming the denominator nonzero, this can also be rewritten into (also see [40], Section 3.6.1):

$$V_{LW}=\frac{V_{X_{1}}V_{X_{2}}}{1-\mu +V_{X_{1}}V_{X_{2}}}$$ (9)

For the example simulation, it was set **μ** = 1; therefore (8) is equivalent to

$$V_{X_{1}}=0\;\text{or}\;V_{X_{2}}=0\;\text{or}\;V_{LW}=1$$ (10)

In the simulation, at *t* = 800 all **LW**-states have value 1 in a precision of 15 digits (and the values *V*_*X*1_, and *V*_*X*2_ are always nonzero). Therefore, for all **LW**-states criterion (6) is fulfilled with deviations < 10^−15^. This provides one piece of evidence that the implemented network model is correct with respect to its design specification.

The 17 **CIW**-states use the combination function **alogistic**_**10,0.4**_**(‥)** described by (4) and the weight of the connection from the related **LW**-state to the **CIW**-state is 1 so

$${\text{aggimpact}}_{CIW}=alogistic_{10,0.4}\left(V_{LW}\right)$$ (11)

where *V*_**LW**_ is the value of the **LW**

Therefore, for this case the equilibrium equation from criterion (6) is

$$alogistic_{10,0.4}\left(V_{LW}\right)=V_{CIW}$$ (12)

where *V*_**CIW**_ is the value of the **CIW**-state.

Now, as already found above at *t* = 800, for all **LW**-states *V*_**LW**_ = 1 in a precision of 15 digits, and **alogistic**_**10,0.4**_(1) = 0.997482089170521. Moreover, at *t* = 800 it is found that *V*_**CIW**_ = 0.997482089170520 for all **CIW**-states. This makes a deviation of 0.997482089170520–0.997482089170521 = -10^−15^. This very small deviation provides a second piece of evidence that the implemented network model is correct with respect to its design specification.

### 6.3 Equilibrium analysis of the IW-states

The **IW**-states use the combination function **alogistic**_**10,0.7**_**(‥)** described by (4) and has incoming connections from themselves (with weight 1), and from the related **IS**-state. Moreover, the connection from this **IS**-state to the **IW**-state has weight represented by the related **CIW**-state, whereas the state values of the **IS**-states are constant 1. Therefore, it holds

$${\text{aggimpact}}_{IW}=alogistic_{10,0.7}\left(V_{IW},V_{CIW}\right)$$ (13)

where *V*_**IW**_ is the value of the **IW**-state itself and *V*_**CIW**_ is the value of the **CIW**-state.

So, for this case the equilibrium equation from criterion (6) is

$$alogistic_{10,0.7}\left(V_{IW},V_{CIW}\right)=V_{IW}$$ (14)

These values have been computed (independent of the implemented model), as shown in Table 7. Here the second and fifth column display the values for the **CIW-** and **IW**-state from the simulation at *t* = 800, and the values in the third and fourth columns were calculated based on that. The fourth column indicates the left hand side of the above Eq (14), the fifth column the right hand side and the sixth column the difference between the two. It turns out that all deviations are < 10^−7^, which is a third piece of evidence that the implemented network model is correct with respect to its design specification.

**Table 7:** Equilibrium analysis results for the IW-states.

nr	*V*_CIW_	*V*_CIW_ + *V*_IW_	aggimpact_IW_	*V*_IW_	deviation
X_29_	0.997482089170520	1.997479769043310	0.999997679872790	0.999997679872789	-1.11022 10^−15^
X_32_	0.997482089170520	1.997479769043310	0.999997679872790	0.999997679872789	-1.11022 10^−15^
X_35_	0.997482089170520	1.997479769043310	0.999997679872790	0.999997679872789	-1.11022 10^−15^
X_38_	0.997482089170520	1.997479769043310	0.999997679872790	0.999997679872789	-1.11022 10^−15^
X_41_	0.997482089170520	1.997479769043310	0.999997679872790	0.999997679872789	-1.11022 10^−15^
X_44_	0.997482089170520	1.997479769043310	0.999997679872790	0.999997679872789	-1.11022 10^−15^
X_47_	0.997482089170520	1.997479769043310	0.999997679872790	0.999997679872789	-1.11022 10^−15^
X_50_	0.997482089170520	1.997479769043310	0.999997679872790	0.999997679872789	-1.11022 10^−15^
X_53_	0.997482089170520	1.997479769043310	0.999997679872790	0.999997679872789	-1.11022 10^−15^
X_56_	0.997482089170520	1.997479769043310	0.999997679872790	0.999997679872789	-1.11022 10^−15^
X_59_	0.997482089170520	1.997479769043310	0.999997679872790	0.999997679872789	-1.11022 10^−15^
X_62_	0.997482089170520	1.997479769043310	0.999997679872790	0.999997679872789	-1.11022 10^−15^
X_65_	0.997482089170520	1.997479769026860	0.999997679872790	0.999997679856344	-1.64454 10^−11^
X_68_	0.997482089170520	1.997479769036000	0.999997679872790	0.999997679865477	-7.31293 10^−12^
X_71_	0.997482089170520	1.997479769029510	0.999997679872790	0.999997679858985	-1.38045 10^−11^
X_74_	0.997482089170520	1.997479763727770	0.999997679872667	0.999997674557245	-5.31542 10^−9^
X_77_	0.997482089170520	1.997479758743110	0.999997679872551	0.999997669572591	-1.03 10^−8^

### 6.4 Equilibrium analysis of the RW-states

The **RW**-states use the combination function **eucl**_**1,2**_**(.,.)** described by (3), which makes the average of its two arguments. They have incoming connections with weight 1 from the related **LW**-state and **IW**-state. Therefore it holds

$${\text{aggimpact}}_{RW}=\frac{V_{LW}+V_{IW}}{2}$$ (15)

where *V*_**LW**_ is the value of the **LW**-state and *V*_**IW**_ is the value of the **IW**-state.

Then for this case the equilibrium equation from criterion (6) is

$$\frac{V_{LW}+V_{IW}}{2}=V_{RW}$$ (16)

where *V*_**RW**_ is the value of the **RW**-state. Like above, these values have been computed (independent of the implemented model) from the simulation values at *t* = 800 for the **IW** and **LW**-states, and compared to the simulation values of the **RW**-states as shown in Table 8. It turns out that all deviations are < 10^−8^, which is a fourth piece of evidence that the implemented network model is correct with respect to its design specification.

**Table 8:** Equilibrium analysis results for the RW-states.

nr	*V*_IW_	*V*_LW_	aggimpact_RW_	*V*_RW_	deviation
X_31_	0.999997679872789	1.000000000000000	0.999998839936394	0.999998839936394	<10^−15^
X_34_	0.999997679872789	1.000000000000000	0.999998839936394	0.999998839936394	<10^−15^
X_37_	0.999997679872789	1.000000000000000	0.999998839936394	0.999998839936394	<10^−15^
X_40_	0.999997679872789	1.000000000000000	0.999998839936394	0.999998839936394	<10^−15^
X_43_	0.999997679872789	1.000000000000000	0.999998839936394	0.999998839936394	<10^−15^
X_46_	0.999997679872789	1.000000000000000	0.999998839936394	0.999998839936394	<10^−15^
X_49_	0.999997679872789	1.000000000000000	0.999998839936394	0.999998839936394	<10^−15^
X_52_	0.999997679872789	1.000000000000000	0.999998839936394	0.999998839936394	<10^−15^
X_55_	0.999997679872789	1.000000000000000	0.999998839936394	0.999998839936394	<10^−15^
X_58_	0.999997679872789	1.000000000000000	0.999998839936394	0.999998839936394	<10^−15^
X_61_	0.999997679872789	1.000000000000000	0.999998839936394	0.999998839936394	<10^−15^
X_64_	0.999997679872789	1.000000000000000	0.999998839936394	0.999998839936394	<10^−15^
X_67_	0.999997679856344	1.000000000000000	0.999998839928172	0.999998839925431	-2.74081 10^−12^
X_70_	0.999997679865477	1.000000000000000	0.999998839932738	0.999998839931520	-1.21869 10^−12^
X_73_	0.999997679858985	1.000000000000000	0.999998839929493	0.999998839927192	-2.30072 10^−12^
X_76_	0.999997674557245	1.000000000000000	0.999998837278622	0.999998836392726	-8.85897 10^−10^
X_79_	0.999997669572591	1.000000000000000	0.999998834786295	0.999998833069649	-1.71665 10^−9^

## 7 Discussion

In this paper, a computational network model was presented for controlled learning of a mental model. Learning of a mental model often involves observational learning and instructional learning. To obtain an effective learning process, appropriate timing of these types of learning is needed, which requires some form of control. For such control, a mental model adaptation process itself has to be made adaptive as well, which is a form of second-order adaptation for this mental model. So, all in all, a mental model can be used in three different manners: (1) it is executed to draw conclusions from it, (2) it is adapted to learn and improve it, and (3) these adaptation processes are controlled. These three properties, and their interplay require three different types of modeling that interact with each other. In this paper, the network-oriented modeling approach for self-modeling adaptive networks described in [23] was applied to address these processes for mental models. A generic three-level self-modeling network architecture introduced in [26] was applied to support this. Based on this general architecture, a second-order adaptive mental network model was presented, in which the base level includes a mental model as it can be used, the second level models a first-order adaptation process for the learning process of this mental model and the third level models a second-order adaptation process that controls the focus and timing of the types of learning.

It has turned out that the network self-modeling mechanism (also called network reification) fits very well to what is needed for (1), (2) and (3) for mental models. The idea of self-modeling networks was originally (in [22, 23]) mainly inspired by the extensive neuroscience literature on plasticity versus metaplasticity in the brain; e.g., [47–50]. Paper [26] was the first paper that demonstrated the usefulness of the same conceptualisation for the social domain of teaching and learning. As far as the authors know, there is no other computational model covering (1), (2) and (3).

The introduced network model was illustrated for a case study of learner-controlled mental model learning for how a car works, and driving it. Here the learner is in control of the use and timing of observational learning and instructional learning. Using the dedicated software environment described in [40], Ch. 9 the network model was implemented and simulated. By this it was shown to work as expected from the literature. Moreover, by verification of the implemented model based on equilibrium analysis (for a representative test set of 68 of the 113 network states), it was found that all deviations are <10^−7^ (see Section 6). This provides strong evidence that the implemented model is correct with respect to its design specification. Further validation by comparison to empirical data would be interesting for future research; currently, such data are not available to the authors.

Much literature exists which describes the learning of mental models and was discussed in the paper. However, computational models addressing it are very rare; a few exceptions are [13, 19, 20, 53]. For example [20], addresses simulation of students’ construction of energy models in physics in a production rule modeling format and in [13] the PDP modeling format was applied to model mental models. In [53] a mental God model was addressed, and in [19] the focus is on model-based learning to drive a car. In all four cases [13, 19, 20, 53] no control of the learning processes is modeled, which is a main difference with the current paper, where the focus is on the control and this is addressed by designing a second-order adaptive mental network model.

In the meantime, the self-modeling network modeling perspective [22, 23] and the general three-level second-order adaptive network architecture introduced in [26] and also described in more detail in the current paper to model dynamics, adaptation of control for mental models, has been found to be very general and applicable for handling mental models in many other application cases where mental models are used. For example, in [54] this architecture and also the learning mechanisms contributed by [26] have been applied successfully to model how shared mental models are used in hospital teamwork. A more detailed overview of this general approach to mental models originating to a large extent in [26] and many of its applications will be presented in the forthcoming book [55].

However, note that the literature on mental models is very diverse. Therefore, although having turned out applicable in many cases, it cannot be claimed that the way in which mental models are addressed from a network-oriented perspective here, would be applicable for all forms of mental models addressed in the literature.

## Supporting information

S1 Appendix Full specification of the second-order adaptive network model. (DOCX)
