# Supplementary material for: Modeling learner-controlled mental model learning processes by a second-order adaptive network model
Source: PLoS One. 2021 Aug 24;16(8):e0255503. doi: 10.1371/journal.pone.0255503 (PMC8384201; doi:10.1371/journal.pone.0255503)
Supplement: S1 Appendix — (DOCX) [file pone.0255503.s001.docx]

Appendix: Full specification of the second-order adaptive network model

The specification of the network model by role matrices

Role matrices provide an overview of the different types of factors that causally affect the network states. In each of the role matrices, each state X*_i_* in the network has its own row where it is listed which other states or characteristics affect this state from that role. For example, in (base) role matrix **mb** it is indicated which other states affect a given state X*_i_* (because there are incoming connections from them), while in role matrix **mcw** (for connection weights) it is indicated what are the connection weights of these connections. Together, these role matrices **mb** and **mcw** define the *connectivity characteristics* of the network model. Moreover, in role matrix **mcfw** (for combination function weights) it is indicated how a given state X*_i_* is affected by the choice of combination function(s) made for this state, while in role matrix **mcfp** (for combination function parameters) the parameters for these combination functions are indicated. Together, role matrices **mcfw** and **mcfp** define the *aggregation characteristics* of the network model. Finally, the *timing characteristics* of the network model are defined by role matrix **ms** (for speed factors). In the nonempty cells in role matrices there is either a static value or a pointer (reference) to a state that represents this value in a dynamic manner as a self-model state. The latter option is the detailed specification of what in the 3D pictures are the pink downward arrows. It specifies the specific role of the causal effect; this provides a quite compact specification of the different self-model levels. For further explanation of the concept of role matrices, see[23], Ch. 9.

As can be seen, the values of all these network characteristics are relatively straightforward. For example, practically all connection weights have value 1. Furthermore, it is assumed that mental model BS-states change much faster than OS- and **IW**-states involving observation of the world or communication from an instructor. Therefore the mental states have speed factors 0.4, whereas the states involving observation or communication have speed factors 0.05. Other values can work as well; small changes of the values used do not really affect the patterns that are generated by the simulations. Basically, the states change values from 0 to 1 in a temporal order that is generated by the causal relations between the world (observation) OS-states and, accordingly, following Craik [2], p. 51, the corresponding causal relations between the mental model BS-states. These 0-to-1 transitions make the model quite robust.

| **mb base-connectivity** | | **1** | **2** | **3** | **4** |
| --- | --- | --- | --- | --- | --- |
| X_1_ | BS_Switch_ | X_1_ | X_15_ |  |  |
| X_2_ | BS_TurnSwitch_ | X_1_ | X_16_ |  |  |
| X_3_ | BS_Engine-0n_ | X_2_ | X_11_ | X_17_ |  |
| X_4_ | BS_FeelEngine-On_ | X_3_ | X_18_ |  |  |
| X_5_ | BS_PresClutch_ | X_4_ | X_19_ |  |  |
| X_6_ | BS_Clutch-On_ | X_5_ | X_20_ |  |  |
| X_7_ | BS_Gearbox-Neutral_ | X_6_ | X _21_ |  |  |
| X_8_ | BS_PressGear 1_ | X_7_ | X_22_ |  |  |
| X_9_ | BS_Gear1-On_ | X_8_ | X_23_ |  |  |
| X_10_ | BS_PressAccelerator_ | X_9_ | X_13_ | X_24_ |  |
| X_11_ | BS_Accelerator-On_ | X_10_ | X_25_ |  |  |
| X_12_ | BS_RevMeter-On_ | X_3_ | X_26_ |  |  |
| X_13_ | BSe_BiteState_ | X _6_ | X_12_ | X_27_ |  |
| X_14_ | BS_MovingState_ | X _3_ | X_9_ | X_28_ |  |
| X_15_ | OS_Switch_ | X_15_ |  |  |  |
| X_16_ | OS_TurnSwitch_ | X_15_ |  |  |  |
| X_17_ | OS_Engine-0n_ | X_16_ | X_25_ |  |  |
| X_18_ | OS_FeelEngine-On_ | X_17_ |  |  |  |
| X_19_ | OS_PressClutch_ | X_18_ | X_24_ |  |  |
| X_20_ | OS_Clutch-On_ | X_19_ |  |  |  |
| X_21_ | OS_Gearbox-Neutral_ | X_20_ |  |  |  |
| X_22_ | OS_PressGear 1_ | X_21_ |  |  |  |
| X_23_ | OS_Gear1_ | X_22_ |  |  |  |
| X_24_ | OS_PressAccelerator_ | X_19_ | X_23_ | X_27_ |  |
| X_25_ | OS_Accelerator-On_ | X_24_ |  |  |  |
| X_26_ | OS_RevMeter-On_ | X_25_ |  |  |  |
| X_27_ | OS_BiteState_ | X_19_ | X_26_ |  |  |
| X_28_ | OS_MovingState_ | X_17_ | X_23_ |  |  |
| X_29_ | IW _Switch,TurnSwitch_ | X_29_ | X_80_ |  |  |
| X_30_ | LW _Switch,TurnSwitch_ | X_1_ | X_2_ | X_30_ |  |
| X_31_ | RW _Switch,TurnSwitch_ | X_29_ | X_30_ |  |  |
| X_32_ | IW _TurnSwitch,Engine-On_ | X_32_ | X_81_ |  |  |
| X_33_ | LW _TurnSwitch,Engine-On_ | X_2_ | X_3_ | X_33_ |  |
| X_34_ | RW _TurnSwitch,Engine-On_ | X_32_ | X_33_ |  |  |
| X_35_ | IW _Engine-On,FeelEngine-On_ | X_35_ | X_82_ |  |  |
| X_36_ | LW _Engine-On,FeelEngine-On_ | X_3_ | X_4_ | X_36_ |  |
| X_37_ | RW _Engine-On,FeelEngine-On_ | X_35_ | X_36_ |  |  |
| X_38_ | IW _FeelEngine-On,PressClutch_ | X_38_ | X_83_ |  |  |
| X_39_ | LW _FeelEngine-On,PressClutch_ | X_4_ | X_5_ | X_39_ |  |
| X_40_ | RW _FeelEngine-On,PressClutch_ | X_38_ | X_39_ |  |  |
| X_41_ | IW _PressClutch,Clutch-On_ | X_41_ | X_84_ |  |  |
| X_42_ | LW _PressClutch,Clutch-On_ | X_5_ | X_6_ | X_42_ |  |
| X_43_ | RW _PressClutch,Clutch-On_ | X_41_ | X_42_ |  |  |
| X_44_ | IW _Clutch-On,Gearbox-Neutral_ | X_44_ | X_85_ |  |  |
| X_45_ | LW _Clutch-On,Gearbox-Neutral_ | X_5_ | X_10_ | X_45_ |  |
| X_46_ | RW _Clutch-On,Gearbox-Neutral_ | X_44_ | X_45_ |  |  |
| X_47_ | IW _Gearbox-Neutral,PressGear1_ | X_47_ | X_86_ |  |  |
| X_48_ | LW _Gearbox-Neutral,PressGear1_ | X_6_ | X_7_ | X_48_ |  |
| X_49_ | RW _Gearbox-Neutral,PressGear1_ | X_47_ | X_48_ |  |  |
| X_50_ | IW _PressGear1,Gear1-On_ | X_50_ | X_87_ |  |  |
| X_51_ | LW _PressGear1,Gear1-On_ | X_7_ | X_8_ | X_51_ |  |
| X_52_ | RW _PressGear1,Gear1-On_ | X_50_ | X_51_ |  |  |
| X_53_ | IW _Gear1-On,PressAccelerator_ | X_53_ | X_88_ |  |  |
| X_54_ | LW _Gear1-On,PressAccelerator_ | X_8_ | X_9_ | X_54_ |  |
| X_55_ | RW _Gear1-On,PressAccelerator_ | X_53_ | X_54_ |  |  |
| X_56_ | IW _PressAccelerator,Accelerator-On_ | X_56_ | X_89_ |  |  |
| X_57_ | LW _PressAccelerator,Accelerator-On_ | X _9_ | X_10_ | X_57_ |  |
| X_58_ | RW _PressAccelerator,Accelerator-On_ | X_56_ | X_57_ |  |  |
| X_59_ | IW _Accelerator-On,Engine-On_ | X_59_ | X_90_ |  |  |
| X_60_ | LW _Accelerator-On,Engine-On_ | X_10_ | X_11_ | X_60_ |  |
| X_61_ | RW _Accelerator-On,Engine-On_ | X_59_ | X_60_ |  |  |
| X_62_ | IW _Engine-On,Rev-Meter-On_ | X_62_ | X_91_ |  |  |
| X_63_ | LW _Engine-On,Rev-Meter-On_ | X_10_ | X_5_ | X_63_ |  |
| X_64_ | RW _Engine-On,Rev-Meter-On_ | X_62_ | X_63_ |  |  |
| X_65_ | IW _RevMeter-On,BiteState_ | X_65_ | X_92_ |  |  |
| X_66_ | LW _RevMeter-On,BiteState_ | X_11_ | X_3_ | X_66_ |  |
| X_67_ | RW _RevMeter-On,BiteState_ | X_65_ | X_66_ |  |  |
| X_68_ | IW _Clutch-On,BiteState_ | X_68_ | X_93_ |  |  |
| X_69_ | LW _Clutch-On,BiteState_ | X_3_ | X_12_ | X_69_ |  |
| X_70_ | RW _Clutch-On,BiteState_ | X_68_ | X_69_ |  |  |
| X_71_ | IW _BiteState,PressAccelerator_ | X_71_ | X_94_ |  |  |
| X_72_ | LW _BiteState,PressAccelerator_ | X_12_ | X_13_ | X_72_ |  |
| X_73_ | RW _BiteState,PressAccelerator_ | X_71_ | X_72_ |  |  |
| X_74_ | IW _Engine-On,MovingState_ | X_74_ | X_95_ |  |  |
| X_75_ | LW _Engine-On,MovingState_ | X _6_ | X_13_ | X_75_ |  |
| X_76_ | RW _Engine-On,MovingState_ | X_74_ | X_75_ |  |  |
| X_77_ | IW _Gear1-On,MovingState_ | X_77_ | X_96_ |  |  |
| X_78_ | LW _Gear1-On,MovingState_ | X_10_ | X_13_ | X_78_ |  |
| X_79_ | RW _Gear1-On,MovingState_ | X_77_ | X_78_ |  |  |
| X_80_ | IS _Switch,TurnSwitch_ | X_80_ |  |  |  |
| X_81_ | IS _TurnSwitch,Engine-On_ | X_3_ |  |  |  |
| X_82_ | IS _Engine-On,FeelEngine-On_ | X_80_ |  |  |  |
| X_83_ | IS _FeelEngineOn,PressClutch_ | X_83_ |  |  |  |
| X_84_ | IS _PressClutch,Clutch-On_ | X_9_ |  |  |  |
| X_85_ | IS _Clutch-On,Gear-BoxNeutral_ | X_83_ |  |  |  |
| X_86_ | IS _GearBox-Neutral,PressGear1_ | X_86_ |  |  |  |
| X_87_ | IS _PressGear1,Gear1-On_ | X_87_ |  |  |  |
| X_88_ | IS _Gear1-On, PressAccelerator_ | X_88_ |  |  |  |
| X_89_ | IS _PressAccelerator, Accelerator-On_ | X_89_ |  |  |  |
| X_90_ | IS _Accelerator-On, Engine-On_ | X_90_ |  |  |  |
| X_91_ | IS _Engine-On,Rev-Meter-On_ | X_91_ |  |  |  |
| X_92_ | IS _RevMeter-On,BiteState_ | X_92_ |  |  |  |
| X_93_ | IS _Clutch-On,BiteState_ | X_93_ |  |  |  |
| X_94_ | IS _BiteState,PressAccelerator_ | X_94_ |  |  |  |
| X_95_ | IS _Engine-On,MovingState_ | X_95_ |  |  |  |
| X_96_ | IS _Gear-On1,MovingState_ | X_96_ |  |  |  |
| X_97_ | CIW _Switch,TurnSwitch_ | X_30_ |  |  |  |
| X_98_ | CIW _TurnSwitch,Engine-On_ | X_33_ |  |  |  |
| X_99_ | CIW _Engine-On,FeelEngine-On_ | X_36_ |  |  |  |
| X_100_ | CIW _FeelEngineOn,PressClutch_ | X_39_ |  |  |  |
| X_101_ | CIW _PressClutch,Clutch-On_ | X_42_ |  |  |  |
| X_102_ | CIW _Clutch-On,Gear-BoxNeutral_ | X_45_ |  |  |  |
| X_103_ | CIW _GearBox-Neutral,PressGear1_ | X_48_ |  |  |  |
| X_104_ | CIW _PressGear1,Gear1-On_ | X_51_ |  |  |  |
| X_105_ | CIW _Gear1-On, PressAccelerator_ | X_54_ |  |  |  |
| X_106_ | CIW _PressAccelerator, Accelerator-On_ | X_57_ |  |  |  |
| X_107_ | CIW _Accelerator-On, Engine-On_ | X_60_ |  |  |  |
| X_108_ | CIW _Engine-On,Rev-Meter-On_ | X_63_ |  |  |  |
| X_109_ | CIW _RevMeter-On,BiteState_ | X_66_ |  |  |  |
| X_110_ | CIW _Clutch-On,BiteState_ | X_69_ |  |  |  |
| X_111_ | CIW _BiteState,PressAccelerator_ | X_72_ |  |  |  |
| X_112_ | CIW _Engine-On,MovingState_ | X_75_ |  |  |  |
| X_113_ | CIW _Gear-On1,MovingState_ | X_78_ |  |  |  |

| **mcw connection weights** | | **1** | **2** | **3** | **4** |
| --- | --- | --- | --- | --- | --- |
| X_1_ | BS_Switch_ | 1 | 1 |  |  |
| X_2_ | BS_TurnSwitch_ | X_31_ | 1 |  |  |
| X_3_ | BS_Engine-0n_ | X_34_ | X_61_ | 1 |  |
| X_4_ | BS_FeelEngine-On_ | X_37_ | 0.5 |  |  |
| X_5_ | BS_PresClutch_ | X_40_ | 0.5 |  |  |
| X_6_ | BS_Clutch-On_ | X_43_ | 0.5 |  |  |
| X_7_ | BS_Gearbox-Neutral_ | X_46_ | 1 |  |  |
| X_8_ | BS_PressGear 1_ | X_49_ | 0 |  |  |
| X_9_ | BS_Gear1-On_ | X_52_ | 0.5 |  |  |
| X_10_ | BS_PressAccelerator_ | X_55_ | X_73_ | 0.3 |  |
| X_11_ | BS_Accelerator-On_ | X_58_ | 0.5 |  |  |
| X_12_ | BS_RevMeter-On_ | X_64_ | 0.4 |  |  |
| X_13_ | BSe_BiteState_ | X_67_ | X_70_ | 0.5 |  |
| X_14_ | BS_MovingState_ | X_76_ | X_79_ | 0.5 |  |
| X_15_ | OS_Switch_ | 1 |  |  |  |
| X_16_ | OS_TurnSwitch_ | 1 |  |  |  |
| X_17_ | OS_Engine-0n_ | 1 | 1 |  |  |
| X_18_ | OS_FeelEngine-On_ | 1 |  |  |  |
| X_19_ | OS_PressClutch_ | 1 |  |  |  |
| X_20_ | OS_Clutch-On_ | 1 |  |  |  |
| X_21_ | OS_Gearbox-Neutral_ | 1 |  |  |  |
| X_22_ | OS_PressGear 1_ | 1 |  |  |  |
| X_23_ | OS_Gear1_ | 1 |  |  |  |
| X_24_ | OS_PressAccelerator_ | 1 | 1 |  |  |
| X_25_ | OS_Accelerator-On_ | 1 |  |  |  |
| X_26_ | OS_RevMeter-On_ | 1 |  |  |  |
| X_27_ | OS_BiteState_ | 1 | 1 |  |  |
| X_28_ | OS_MovingState_ | 1 | 1 |  |  |
| X_29_ | IW _Switch,TurnSwitch_ | 1 | X_97_ |  |  |
| X_30_ | LW _Switch,TurnSwitch_ | 1 | 1 | 1 |  |
| X_31_ | RW _Switch,TurnSwitch_ | 1 | 1 |  |  |
| X_32_ | IW _TurnSwitch,Engine-On_ | 1 | X_98_ |  |  |
| X_33_ | LW _TurnSwitch,Engine-On_ | 1 | 1 | 1 |  |
| X_34_ | RW _TurnSwitch,Engine-On_ | 1 | 1 |  |  |
| X_35_ | IW _Engine-On,FeelEngine-On_ | 1 | X_99_ |  |  |
| X_36_ | LW _Engine-On,FeelEngine-On_ | 1 | 1 | 1 |  |
| X_37_ | RW _Engine-On,FeelEngine-On_ | 1 | 1 |  |  |
| X_38_ | IW _FeelEngine-On,PressClutch_ | 1 | X_100_ |  |  |
| X_39_ | LW _FeelEngine-On,PressClutch_ | 1 | 1 | 1 |  |
| X_40_ | RW _FeelEngine-On,PressClutch_ | 1 | 1 |  |  |
| X_41_ | IW _PressClutch,Clutch-On_ | 1 | X_101_ |  |  |
| X_42_ | LW _PressClutch,Clutch-On_ | 1 | 1 | 1 |  |
| X_43_ | RW _PressClutch,Clutch-On_ | 1 | 1 |  |  |
| X_44_ | IW _Clutch-On,Gearbox-Neutral_ | 1 | X_102_ |  |  |
| X_45_ | LW _Clutch-On,Gearbox-Neutral_ | 1 | 1 | 1 |  |
| X_46_ | RW _Clutch-On,Gearbox-Neutral_ | 1 | 1 |  |  |
| X_47_ | IW _Gearbox-Neutral,PressGear1_ | 1 | X_103_ |  |  |
| X_48_ | LW _Gearbox-Neutral,PressGear1_ | 1 | 1 | 1 |  |
| X_49_ | RW _Gearbox-Neutral,PressGear1_ | 1 | 1 |  |  |
| X_50_ | IW _PressGear1,Gear1-On_ | 1 | X_104_ |  |  |
| X_51_ | LW _PressGear1,Gear1-On_ | 1 | 1 | 1 |  |
| X_52_ | RW _PressGear1,Gear1-On_ | 1 | 1 |  |  |
| X_53_ | IW _Gear1-On,PressAccelerator_ | 1 | X_105_ |  |  |
| X_54_ | LW _Gear1-On,PressAccelerator_ | 1 | 1 | 1 |  |
| X_55_ | RW _Gear1-On,PressAccelerator_ | 1 | 1 |  |  |
| X_56_ | IW _PressAccelerator,Accelerator-On_ | 1 | X_106_ |  |  |
| X_57_ | LW _PressAccelerator,Accelerator-On_ | 1 | 1 | 1 |  |
| X_58_ | RW _PressAccelerator,Accelerator-On_ | 1 | 1 |  |  |
| X_59_ | IW _Accelerator-On,Engine-On_ | 1 | X_107_ |  |  |
| X_60_ | LW _Accelerator-On,Engine-On_ | 1 | 1 | 1 |  |
| X_61_ | RW _Accelerator-On,Engine-On_ | 1 | 1 |  |  |
| X_62_ | IW _Engine-On,Rev-Meter-On_ | 1 | X_108_ |  |  |
| X_63_ | LW _Engine-On,Rev-Meter-On_ | 1 | 1 | 1 |  |
| X_64_ | RW _Engine-On,Rev-Meter-On_ | 1 | 1 |  |  |
| X_65_ | IW _RevMeter-On,BiteState_ | 1 | X_109_ |  |  |
| X_66_ | LW _RevMeter-On,BiteState_ | 1 | 1 | 1 |  |
| X_67_ | RW _RevMeter-On,BiteState_ | 1 | 1 |  |  |
| X_68_ | IW _Clutch-On,BiteState_ | 1 | X_110_ |  |  |
| X_69_ | LW _Clutch-On,BiteState_ | 1 | 1 | 1 |  |
| X_70_ | RW _Clutch-On,BiteState_ | 1 | 1 |  |  |
| X_71_ | IW _BiteState,PressAccelerator_ | 1 | X_111_ |  |  |
| X_72_ | LW _BiteState,PressAccelerator_ | 1 | 1 | 1 |  |
| X_73_ | RW _BiteState,PressAccelerator_ | 1 | 1 |  |  |
| X_74_ | IW _Engine-On,MovingState_ | 1 | X_112_ |  |  |
| X_75_ | LW _Engine-On,MovingState_ | 1 | 1 | 1 |  |
| X_76_ | RW _Engine-On,MovingState_ | 1 | 1 |  |  |
| X_77_ | IW _Gear1-On,MovingState_ | 1 | X_113_ |  |  |
| X_78_ | LW _Gear1-On,MovingState_ | 1 | 1 | 1 |  |
| X_79_ | RW _Gear1-On,MovingState_ | 1 | 1 |  |  |
| X_80_ | IS _Switch,TurnSwitch_ | 1 |  |  |  |
| X_81_ | IS _TurnSwitch,Engine-On_ | 1 |  |  |  |
| X_82_ | IS _Engine-On,FeelEngine-On_ | 1 |  |  |  |
| X_83_ | IS _FeelEngineOn,PressClutch_ | 1 |  |  |  |
| X_84_ | IS _PressClutch,Clutch-On_ | 1 |  |  |  |
| X_85_ | IS _Clutch-On,Gear-BoxNeutral_ | 1 |  |  |  |
| X_86_ | IS _GearBox-Neutral,PressGear1_ | 1 |  |  |  |
| X_87_ | IS _PressGear1,Gear1-On_ | 1 |  |  |  |
| X_88_ | IS _Gear1-On, PressAccelerator_ | 1 |  |  |  |
| X_89_ | IS _PressAccelerator, Accelerator-On_ | 1 |  |  |  |
| X_90_ | IS _Accelerator-On, Engine-On_ | 1 |  |  |  |
| X_91_ | IS _Engine-On,Rev-Meter-On_ | 1 |  |  |  |
| X_92_ | IS _RevMeter-On,BiteState_ | 1 |  |  |  |
| X_93_ | IS _Clutch-On,BiteState_ | 1 |  |  |  |
| X_94_ | IS _BiteState,PressAccelerator_ | 1 |  |  |  |
| X_95_ | IS _Engine-On,MovingState_ | 1 |  |  |  |
| X_96_ | IS _Gear-On1,MovingState_ | 1 |  |  |  |
| X_97_ | CIW _Switch,TurnSwitch_ | 1 |  |  |  |
| X_98_ | CIW _TurnSwitch,Engine-On_ | 1 |  |  |  |
| X_99_ | CIW _Engine-On,FeelEngine-On_ | 1 |  |  |  |
| X_100_ | CIW _FeelEngineOn,PressClutch_ | 1 |  |  |  |
| X_101_ | CIW _PressClutch,Clutch-On_ | 1 |  |  |  |
| X_102_ | CIW _Clutch-On,Gear-BoxNeutral_ | 1 |  |  |  |
| X_103_ | CIW _GearBox-Neutral,PressGear1_ | 1 |  |  |  |
| X_104_ | CIW _PressGear1,Gear1-On_ | 1 |  |  |  |
| X_105_ | CIW _Gear1-On, PressAccelerator_ | 1 |  |  |  |
| X_106_ | CIW _PressAccelerator, Accelerator-On_ | 1 |  |  |  |
| X_107_ | CIW _Accelerator-On, Engine-On_ | 1 |  |  |  |
| X_108_ | CIW _Engine-On,Rev-Meter-On_ | 1 |  |  |  |
| X_109_ | CIW _RevMeter-On,BiteState_ | 1 |  |  |  |
| X_110_ | CIW _Clutch-On,BiteState_ | 1 |  |  |  |
| X_111_ | CIW _BiteState,PressAccelerator_ | 1 |  |  |  |
| X_112_ | CIW _Engine-On,MovingState_ | 1 |  |  |  |
| X_113_ | CIW _Gear-On1,MovingState_ | 1 |  |  |  |

| **ms speed factors** | | **1** |
| --- | --- | --- |
| X_1_ | BS_Switch_ | 0.4 |
| X_2_ | BS_TurnSwitch_ | 0.4 |
| X_3_ | BS_Engine-0n_ | 0.4 |
| X_4_ | BS_FeelEngine-On_ | 0.4 |
| X_5_ | BS_PresClutch_ | 0.4 |
| X_6_ | BS_Clutch-On_ | 0.4 |
| X_7_ | BS_Gearbox-Neutral_ | 0.4 |
| X_8_ | BS_PressGear 1_ | 0.4 |
| X_9_ | BS_Gear1-On_ | 0.4 |
| X_10_ | BS_PressAccelerator_ | 0.4 |
| X_11_ | BS_Accelerator-On_ | 0.4 |
| X_12_ | BS_RevMeter-On_ | 0.4 |
| X_13_ | BSe_BiteState_ | 0.4 |
| X_14_ | BS_MovingState_ | 0.4 |
| X_15_ | OS_Switch_ | 0.05 |
| X_16_ | OS_TurnSwitch_ | 0.05 |
| X_17_ | OS_Engine-0n_ | 0.05 |
| X_18_ | OS_FeelEngine-On_ | 0.05 |
| X_19_ | OS_PressClutch_ | 0.05 |
| X_20_ | OS_Clutch-On_ | 0.05 |
| X_21_ | OS_Gearbox-Neutral_ | 0.05 |
| X_22_ | OS_PressGear 1_ | 0.05 |
| X_23_ | OS_Gear1_ | 0.05 |
| X_24_ | OS_PressAccelerator_ | 0.05 |
| X_25_ | OS_Accelerator-On_ | 0.05 |
| X_26_ | OS_RevMeter-On_ | 0.05 |
| X_27_ | OS_BiteState_ | 0.05 |
| X_28_ | OS_MovingState_ | 0.05 |
| X_29_ | IW _Switch,TurnSwitch_ | 0.1 |
| X_30_ | LW _Switch,TurnSwitch_ | 0.4 |
| X_31_ | RW _Switch,TurnSwitch_ | 0.4 |
| X_32_ | IW _TurnSwitch,Engine-On_ | 0.1 |
| X_33_ | LW _TurnSwitch,Engine-On_ | 0.4 |
| X_34_ | RW _TurnSwitch,Engine-On_ | 0.4 |
| X_35_ | IW _Engine-On,FeelEngine-On_ | 0.1 |
| X_36_ | LW _Engine-On,FeelEngine-On_ | 0.4 |
| X_37_ | RW _Engine-On,FeelEngine-On_ | 0.4 |
| X_38_ | IW _FeelEngine-On,PressClutch_ | 0.1 |
| X_39_ | LW _FeelEngine-On,PressClutch_ | 0.4 |
| X_40_ | RW _FeelEngine-On,PressClutch_ | 0.4 |
| X_41_ | IW _PressClutch,Clutch-On_ | 0.1 |
| X_42_ | LW _PressClutch,Clutch-On_ | 0.4 |
| X_43_ | RW _PressClutch,Clutch-On_ | 0.4 |
| X_44_ | IW _Clutch-On,Gearbox-Neutral_ | 0.1 |
| X_45_ | LW _Clutch-On,Gearbox-Neutral_ | 0.4 |
| X_46_ | RW _Clutch-On,Gearbox-Neutral_ | 0.4 |
| X_47_ | IW _Gearbox-Neutral,PressGear1_ | 0.1 |
| X_48_ | LW _Gearbox-Neutral,PressGear1_ | 0.4 |
| X_49_ | RW _Gearbox-Neutral,PressGear1_ | 0.4 |
| X_50_ | IW _PressGear1,Gear1-On_ | 0.1 |
| X_51_ | LW _PressGear1,Gear1-On_ | 0.4 |
| X_52_ | RW _PressGear1,Gear1-On_ | 0.4 |
| X_53_ | IW _Gear1-On,PressAccelerator_ | 0.1 |
| X_54_ | LW _Gear1-On,PressAccelerator_ | 0.4 |
| X_55_ | RW _Gear1-On,PressAccelerator_ | 0.4 |
| X_56_ | IW _PressAccelerator,Accelerator-On_ | 0.1 |
| X_57_ | LW _PressAccelerator,Accelerator-On_ | 0.4 |
| X_58_ | RW _PressAccelerator,Accelerator-On_ | 0.4 |
| X_59_ | IW _Accelerator-On,Engine-On_ | 0.1 |
| X_60_ | LW _Accelerator-On,Engine-On_ | 0.4 |
| X_61_ | RW _Accelerator-On,Engine-On_ | 0.4 |
| X_62_ | IW _Engine-On,Rev-Meter-On_ | 0.1 |
| X_63_ | LW _Engine-On,Rev-Meter-On_ | 0.4 |
| X_64_ | RW _Engine-On,Rev-Meter-On_ | 0.4 |
| X_65_ | IW _RevMeter-On,BiteState_ | 0.1 |
| X_66_ | LW _RevMeter-On,BiteState_ | 0.4 |
| X_67_ | RW _RevMeter-On,BiteState_ | 0.4 |
| X_68_ | IW _Clutch-On,BiteState_ | 0.1 |
| X_69_ | LW _Clutch-On,BiteState_ | 0.4 |
| X_70_ | RW _Clutch-On,BiteState_ | 0.4 |
| X_71_ | IW _BiteState,PressAccelerator_ | 0.1 |
| X_72_ | LW _BiteState,PressAccelerator_ | 0.4 |
| X_73_ | RW _BiteState,PressAccelerator_ | 0.4 |
| X_74_ | IW _Engine-On,MovingState_ | 0.1 |
| X_75_ | LW _Engine-On,MovingState_ | 0.4 |
| X_76_ | RW _Engine-On,MovingState_ | 0.4 |
| X_77_ | IW _Gear1-On,MovingState_ | 0.1 |
| X_78_ | LW _Gear1-On,MovingState_ | 0.4 |
| X_79_ | RW _Gear1-On,MovingState_ | 0.4 |
| X_80_ | IS _Switch,TurnSwitch_ | 0 |
| X_81_ | IS _TurnSwitch,Engine-On_ | 0 |
| X_82_ | IS _Engine-On,FeelEngine-On_ | 0 |
| X_83_ | IS _FeelEngineOn,PressClutch_ | 0 |
| X_84_ | IS _PressClutch,Clutch-On_ | 0 |
| X_85_ | IS _Clutch-On,Gear-BoxNeutral_ | 0 |
| X_86_ | IS _GearBox-Neutral,PressGear1_ | 0 |
| X_87_ | IS _PressGear1,Gear1-On_ | 0 |
| X_88_ | IS _Gear1-On, PressAccelerator_ | 0 |
| X_89_ | IS _PressAccelerator, Accelerator-On_ | 0 |
| X_90_ | IS _Accelerator-On, Engine-On_ | 0 |
| X_91_ | IS _Engine-On,Rev-Meter-On_ | 0 |
| X_92_ | IS _RevMeter-On,BiteState_ | 0 |
| X_93_ | IS _Clutch-On,BiteState_ | 0 |
| X_94_ | IS _BiteState,PressAccelerator_ | 0 |
| X_95_ | IS _Engine-On,MovingState_ | 0 |
| X_96_ | IS _Gear-On1,MovingState_ | 0 |
| X_97_ | CIW _Switch,TurnSwitch_ | 0.4 |
| X_98_ | CIW _TurnSwitch,Engine-On_ | 0.4 |
| X_99_ | CIW _Engine-On,FeelEngine-On_ | 0.4 |
| X_100_ | CIW _FeelEngineOn,PressClutch_ | 0.4 |
| X_101_ | CIW _PressClutch,Clutch-On_ | 0.4 |
| X_102_ | CIW _Clutch-On,Gear-BoxNeutral_ | 0.4 |
| X_103_ | CIW _GearBox-Neutral,PressGear1_ | 0.4 |
| X_104_ | CIW _PressGear1,Gear1-On_ | 0.4 |
| X_105_ | CIW _Gear1-On, PressAccelerator_ | 0.4 |
| X_106_ | CIW _PressAccelerator, Accelerator-On_ | 0.4 |
| X_107_ | CIW _Accelerator-On, Engine-On_ | 0.4 |
| X_108_ | CIW _Engine-On,Rev-Meter-On_ | 0.4 |
| X_109_ | CIW _RevMeter-On,BiteState_ | 0.4 |
| X_110_ | CIW _Clutch-On,BiteState_ | 0.4 |
| X_111_ | CIW _BiteState,PressAccelerator_ | 0.4 |
| X_112_ | CIW _Engine-On,MovingState_ | 0.4 |
| X_113_ | CIW _Gear-On1,MovingState_ | 0.4 |

| **mcfw combination**  **function**  **weights** | | | | **1** | | | | **2** | | | | **3** | | |  |
| --- | --- | --- | --- | --- | --- | --- | --- | --- | --- | --- | --- | --- | --- | --- | --- |
|  |  |  |  | eucl | | | | alogistic | | | | hebb | | |  |
| X_1_ | | BS_Switch_ | | 1 | | | | |  | |  | | |  |  |
| X_2_ | | BS_TurnSwitch_ | |  | | | | | 1 | |  | | |  |  |
| X_3_ | | BS_Engine-0n_ | |  | | | | | 1 | |  | | |  |  |
| X_4_ | | BS_FeelEngine-On_ | |  | | | | | 1 | |  | | |  |  |
| X_5_ | | BS_PresClutch_ | |  | | | | | 1 | |  | | |  |  |
| X_6_ | | BS_Clutch-On_ | |  | | | | | 1 | |  | | |  |  |
| X_7_ | | BS_Gearbox-Neutral_ | |  | | | | | 1 | |  | | |  |  |
| X_8_ | | BS_PressGear 1_ | |  | | | | | 1 | |  | | |  |  |
| X_9_ | | BS_Gear1-On_ | |  | | | | | 1 | |  | | |  |  |
| X_10_ | | BS_PressAccelerator_ | |  | | | | | 1 | |  | | |  |  |
| X_11_ | | BS_Accelerator-On_ | |  | | | | | 1 | |  | | |  |  |
| X_12_ | | BS_RevMeter-On_ | |  | | | | | 1 | |  | | |  |  |
| X_13_ | | BSe_BiteState_ | |  | | | | | 1 | |  | | |  |  |
| X_14_ | | BS_MovingState_ | |  | | | | | 1 | |  | | |  |  |
| X_15_ | | OS_Switch_ | | 1 | | | | |  | |  | | |  |  |
| X_16_ | | OS_TurnSwitch_ | | 1 | | | | |  | |  | | |  |  |
| X_17_ | | OS_Engine-0n_ | |  | | | | | 1 | |  | | |  |  |
| X_18_ | | OS_FeelEngine-On_ | | 1 | | | | |  | |  | | |  |  |
| X_19_ | | OS_PressClutch_ | | 1 | | | | |  | |  | | |  |  |
| X_20_ | | OS_Clutch-On_ | | 1 | | | | |  | |  | | |  |  |
| X_21_ | | OS_Gearbox-Neutral_ | | 1 | | | | |  | |  | | |  |  |
| X_22_ | | OS_PressGear 1_ | | 1 | | | | |  | |  | | |  |  |
| X_23_ | | OS_Gear1_ | | 1 | | | | |  | |  | | |  |  |
| X_24_ | | OS_PressAccelerator_ | |  | | | | | 1 | |  | | |  |  |
| X_25_ | | OS_Accelerator-On_ | | 1 | | | | |  | |  | | |  |  |
| X_26_ | | OS_RevMeter-On_ | | 1 | | | | |  | |  | | |  |  |
| X_27_ | | OS_BiteState_ | |  | | | | | 1 | |  | | |  |  |
| X_28_ | | OS_MovingState_ | |  | | | | | 1 | |  | | |  |  |
| X_29_ | | IW _Switch,TurnSwitch_ | |  | | | | | 1 | |  | | |  |  |
| X_30_ | | LW _Switch,TurnSwitch_ | |  | | | | |  | | 1 | | |  |  |
| X_31_ | | RW _Switch,TurnSwitch_ | | 1 | | | | |  | |  | | |  |  |
| X_32_ | | IW _TurnSwitch,Engine-On_ | |  | | | | | 1 | |  | | |  |  |
| X_33_ | | LW _TurnSwitch,Engine-On_ | |  | | | | |  | | 1 | | |  |  |
| X_34_ | | RW _TurnSwitch,Engine-On_ | | 1 | | | | |  | |  | | |  |  |
| X_35_ | | IW _Engine-On,FeelEngine-On_ | |  | | | | | 1 | |  | | |  |  |
| X_36_ | | LW _Engine-On,FeelEngine-On_ | |  | | | | |  | | 1 | | |  |  |
| X_37_ | | RW _Engine-On,FeelEngine-On_ | | 1 | | | | |  | |  | | |  |  |
| X_38_ | | IW _FeelEngine-On,PressClutch_ | |  | | | | | 1 | |  | | |  |  |
| X_39_ | | LW _FeelEngine-On,PressClutch_ | |  | | | | |  | | 1 | | |  |  |
| X_40_ | | RW _FeelEngine-On,PressClutch_ | | 1 | | | | |  | |  | | |  |  |
| X_41_ | | IW _PressClutch,Clutch-On_ | |  | | | | | 1 | |  | | |  |  |
| X_42_ | | LW _PressClutch,Clutch-On_ | |  | | | | |  | | 1 | | |  |  |
| X_43_ | | RW _PressClutch,Clutch-On_ | | 1 | | | | |  | |  | | |  |  |
| X_44_ | | IW _Clutch-On,Gearbox-Neutral_ | |  | | | | | 1 | |  | | |  |  |
| X_45_ | | LW _Clutch-On,Gearbox-Neutral_ | |  | | | | |  | | 1 | | |  |  |
| X_46_ | | RW _Clutch-On,Gearbox-Neutral_ | | 1 | | | | |  | |  | | |  |  |
| X_47_ | | IW _Gearbox-Neutral,PressGear1_ | |  | | | | | 1 | |  | | |  |  |
| X_48_ | | LW _Gearbox-Neutral,PressGear1_ | |  | | | | |  | | 1 | | |  |  |
| X_49_ | | RW _Gearbox-Neutral,PressGear1_ | | 1 | | | | |  | |  | | |  |  |
| X_50_ | | IW _PressGear1,Gear1-On_ | |  | | | | | 1 | |  | | |  |  |
| X_51_ | | LW _PressGear1,Gear1-On_ | |  | | | | |  | | 1 | | |  |  |
| X_52_ | | RW _PressGear1,Gear1-On_ | | 1 | | | | |  | |  | | |  |  |
| X_53_ | | IW _Gear1-On,PressAccelerator_ | |  | | | | | 1 | |  | | |  |  |
| X_54_ | | LW _Gear1-On,PressAccelerator_ | |  | | | | |  | | 1 | | |  |  |
| X_55_ | | RW _Gear1-On,PressAccelerator_ | | 1 | | | | |  | |  | | |  |  |
| X_56_ | | IW _PressAccelerator,Accelerator-On_ | |  | | | | | 1 | |  | | |  |  |
| X_57_ | | LW _PressAccelerator,Accelerator-On_ | |  | | | | |  | | 1 | | |  |  |
| X_58_ | | RW _PressAccelerator,Accelerator-On_ | | 1 | | | | |  | |  | | |  |  |
| X_59_ | | IW _Accelerator-On,Engine-On_ | |  | | | | | 1 | |  | | |  |  |
| X_60_ | | LW _Accelerator-On,Engine-On_ | |  | | | | |  | | 1 | | |  |  |
| X_61_ | | RW _Accelerator-On,Engine-On_ | | 1 | | | | |  | |  | | |  |  |
| X_62_ | | IW _Engine-On,Rev-Meter-On_ | |  | | | | | 1 | |  | | |  |  |
| X_63_ | | LW _Engine-On,Rev-Meter-On_ | |  | | | | |  | | 1 | | |  |  |
| X_64_ | | RW _Engine-On,Rev-Meter-On_ | | 1 | | | | |  | |  | | |  |  |
| X_65_ | | IW _RevMeter-On,BiteState_ | |  | | | | | 1 | |  | | |  |  |
| X_66_ | | LW _RevMeter-On,BiteState_ | |  | | | | |  | | 1 | | |  |  |
| X_67_ | | RW _RevMeter-On,BiteState_ | | 1 | | | | |  | |  | | |  |  |
| X_68_ | | IW _Clutch-On,BiteState_ | |  | | | | | 1 | |  | | |  |  |
| X_69_ | | LW _Clutch-On,BiteState_ | |  | | | | |  | | 1 | | |  |  |
| X_70_ | | RW _Clutch-On,BiteState_ | | 1 | | | | |  | |  | | |  |  |
| X_71_ | | IW _BiteState,PressAccelerator_ | |  | | | | | 1 | |  | | |  |  |
| X_72_ | | LW _BiteState,PressAccelerator_ | |  | | | | |  | | 1 | | |  |  |
| X_73_ | | RW _BiteState,PressAccelerator_ | | 1 | | | | |  | |  | | |  |  |
| X_74_ | | IW _Engine-On,MovingState_ | |  | | | | | 1 | |  | | |  |  |
| X_75_ | | LW _Engine-On,MovingState_ | |  | | | | |  | | 1 | | |  |  |
| X_76_ | | RW _Engine-On,MovingState_ | | 1 | | | | |  | |  | | |  |  |
| X_77_ | | IW _Gear1-On,MovingState_ | |  | | | | | 1 | |  | | |  |  |
| X_78_ | | LW _Gear1-On,MovingState_ | |  | | | | |  | | 1 | | |  |  |
| X_79_ | | RW _Gear1-On,MovingState_ | | 1 | | | | |  | |  | | |  |  |
| X_80_ | | IS _Switch,TurnSwitch_ | | 1 | | | | |  | |  | | |  |  |
| X_81_ | | IS _TurnSwitch,Engine-On_ | | 1 | | | | |  | |  | | |  |  |
| X_82_ | | IS _Engine-On,FeelEngine-On_ | | 1 | | | | |  | |  | | |  |  |
| X_83_ | | IS _FeelEngineOn,PressClutch_ | | 1 | | | | |  | |  | | |  |  |
| X_84_ | | IS _PressClutch,Clutch-On_ | | 1 | | | | |  | |  | | |  |  |
| X_85_ | | IS _Clutch-On,Gear-BoxNeutral_ | | 1 | | | | |  | |  | | |  |  |
| X_86_ | | IS _GearBox-Neutral,PressGear1_ | | 1 | | | | |  | |  | | |  |  |
| X_87_ | | IS _PressGear1,Gear1-On_ | | 1 | | | | |  | |  | | |  |  |
| X_88_ | | IS _Gear1-On, PressAccelerator_ | | 1 | | | | |  | |  | | |  |  |
| X_89_ | | IS _PressAccelerator, Accelerator-On_ | | 1 | | | | |  | |  | | |  |  |
| X_90_ | | IS _Accelerator-On, Engine-On_ | | 1 | | | | |  | |  | | |  |  |
| X_91_ | | IS _Engine-On,Rev-Meter-On_ | | 1 | | | | |  | |  | | |  |  |
| X_92_ | | IS _RevMeter-On,BiteState_ | | 1 | | | | |  | |  | | |  |  |
| X_93_ | | IS _Clutch-On,BiteState_ | | 1 | | | | |  | |  | | |  |  |
| X_94_ | | IS _BiteState,PressAccelerator_ | | 1 | | | | |  | |  | | |  |  |
| X_95_ | | IS _Engine-On,MovingState_ | | 1 | | | | |  | |  | | |  |  |
| X_96_ | | IS _Gear-On1,MovingState_ | | 1 | | | | |  | |  | | |  |  |
| X_97_ | | CIW _Switch,TurnSwitch_ | |  | | | | | 1 | |  | | |  |  |
| X_98_ | | CIW _TurnSwitch,Engine-On_ | |  | | | | | 1 | |  | | |  |  |
| X_99_ | | CIW _Engine-On,FeelEngine-On_ | |  | | | | | 1 | |  | | |  |  |
| X_100_ | | CIW _FeelEngineOn,PressClutch_ | |  | | | | | 1 | |  | | |  |  |
| X_101_ | | CIW _PressClutch,Clutch-On_ | |  | | | | | 1 | |  | | |  |  |
| X_102_ | | CIW _Clutch-On,Gear-BoxNeutral_ | |  | | | | | 1 | |  | | |  |  |
| X_103_ | | CIW _GearBox-Neutral,PressGear1_ | |  | | | | | 1 | |  | | |  |  |
| X_104_ | | CIW _PressGear1,Gear1-On_ | |  | | | | | 1 | |  | | |  |  |
| X_105_ | | CIW _Gear1-On, PressAccelerator_ | |  | | | | | 1 | |  | | |  |  |
| X_106_ | | CIW _PressAccelerator, Accelerator-On_ | |  | | | | | 1 | |  | | |  |  |
| X_107_ | | CIW _Accelerator-On, Engine-On_ | |  | | | | | 1 | |  | | |  |  |
| X_108_ | | CIW _Engine-On,Rev-Meter-On_ | |  | | | | | 1 | |  | | |  |  |
| X_109_ | | CIW _RevMeter-On,BiteState_ | |  | | | | | 1 | |  | | |  |  |
| X_110_ | | CIW _Clutch-On,BiteState_ | |  | | | | | 1 | |  | | |  |  |
| X_111_ | | CIW _BiteState,PressAccelerator_ | |  | | | | | 1 | |  | | |  |  |
| X_112_ | | CIW _Engine-On,MovingState_ | |  | | | | | 1 | |  | | |  |  |
| X_113_ | | CIW _Gear-On1,MovingState_ | |  | | | | | 1 | |  | | |  |  |
| **mcfpv combination**  **function**  **parameter values** | | | | 1  eucl | | | 2  alogistic | | | | 3  hebb | | | | |
|  |  |  |  | 1 | | 2 | 1 | 2 | | | 1 | | |  | |
|  |  |  |  | $\boldsymbol{n}$ | | $\boldsymbol{\lambda}$ | $\boldsymbol{\sigma}$ | $\boldsymbol{\tau}$ | | | $\boldsymbol{\mu}$ | | |  | |
| X_1_ | | BS_Switch_ | | 1 | | 2 |  |  | | |  | | |  | |
| X_2_ | | BS_TurnSwitch_ | |  | |  | 10 | 1.2 | | |  | | |  | |
| X_3_ | | BS_Engine-0n_ | |  | |  | 10 | 1.2 | | |  | | |  | |
| X_4_ | | BS_FeelEngine-On_ | |  | |  | 10 | 0.8 | | |  | | |  | |
| X_5_ | | BS_PresClutch_ | |  | |  | 10 | 0.9 | | |  | | |  | |
| X_6_ | | BS_Clutch-On_ | |  | |  | 10 | 0.9 | | |  | | |  | |
| X_7_ | | BS_Gearbox-Neutral_ | |  | |  | 10 | 1.4 | | |  | | |  | |
| X_8_ | | BS_PressGear 1_ | |  | |  | 10 | 0.4 | | |  | | |  | |
| X_9_ | | BS_Gear1-On_ | |  | |  | 10 | 0.9 | | |  | | |  | |
| X_10_ | | BS_PressAccelerator_ | |  | |  | 10 | 0.7 | | |  | | |  | |
| X_11_ | | BS_Accelerator-On_ | |  | |  | 10 | 0.9 | | |  | | |  | |
| X_12_ | | BS_RevMeter-On_ | |  | |  | 12 | 0.97 | | |  | | |  | |
| X_13_ | | BSe_BiteState_ | |  | |  | 10 | 1.2 | | |  | | |  | |
| X_14_ | | BS_MovingState_ | |  | |  | 10 | 1.3 | | |  | | |  | |
| X_15_ | | OS_Switch_ | | 1 | | 1 |  |  | | |  | | |  | |
| X_16_ | | OS_TurnSwitch_ | | 1 | | 1 |  |  | | |  | | |  | |
| X_17_ | | OS_Engine-0n_ | |  | |  | 10 | 0.7 | | |  | | |  | |
| X_18_ | | OS_FeelEngine-On_ | | 1 | | 1 |  |  | | |  | | |  | |
| X_19_ | | OS_PressClutch_ | | 1 | | 1 |  |  | | |  | | |  | |
| X_20_ | | OS_Clutch-On_ | | 1 | | 1 |  |  | | |  | | |  | |
| X_21_ | | OS_Gearbox-Neutral_ | | 1 | | 1 |  |  | | |  | | |  | |
| X_22_ | | OS_PressGear 1_ | | 1 | | 1 |  |  | | |  | | |  | |
| X_23_ | | OS_Gear1_ | | 1 | | 1 |  |  | | |  | | |  | |
| X_24_ | | OS_PressAccelerator_ | |  | |  | 10 | 0.7 | | |  | | |  | |
| X_25_ | | OS_Accelerator-On_ | | 1 | | 1 |  |  | | |  | | |  | |
| X_26_ | | OS_RevMeter-On_ | | 1 | | 1 |  |  | | |  | | |  | |
| X_27_ | | OS_BiteState_ | |  | |  | 10 | 0.7 | | |  | | |  | |
| X_28_ | | OS_MovingState_ | |  | |  | 10 | 0.7 | | |  | | |  | |
| X_29_ | | IW _Switch,TurnSwitch_ | |  | |  | 10 | 0.7 | | |  | | |  | |
| X_30_ | | LW _Switch,TurnSwitch_ | |  | |  |  |  | | | 1 | | |  | |
| X_31_ | | RW _Switch,TurnSwitch_ | | 1 | | 2 |  |  | | |  | | |  | |
| X_32_ | | IW _TurnSwitch,Engine-On_ | |  | |  | 10 | 0.7 | | |  | | |  | |
| X_33_ | | LW _TurnSwitch,Engine-On_ | |  | |  |  |  | | | 1 | | |  | |
| X_34_ | | RW _TurnSwitch,Engine-On_ | | 1 | | 2 |  |  | | |  | | |  | |
| X_35_ | | IW _Engine-On,FeelEngine-On_ | |  | |  | 10 | 0.7 | | |  | | |  | |
| X_36_ | | LW _Engine-On,FeelEngine-On_ | |  | |  |  |  | | | 1 | | |  | |
| X_37_ | | RW _Engine-On,FeelEngine-On_ | | 1 | | 2 |  |  | | |  | | |  | |
| X_38_ | | IW _FeelEngine-On,PressClutch_ | |  | |  | 10 | 0.7 | | |  | | |  | |
| X_39_ | | LW _FeelEngine-On,PressClutch_ | |  | |  |  |  | | | 1 | | |  | |
| X_40_ | | RW _FeelEngine-On,PressClutch_ | | 1 | | 2 |  |  | | |  | | |  | |
| X_41_ | | IW _PressClutch,Clutch-On_ | |  | |  | 10 | 0.7 | | |  | | |  | |
| X_42_ | | LW _PressClutch,Clutch-On_ | |  | |  |  |  | | | 1 | | |  | |
| X_43_ | | RW _PressClutch,Clutch-On_ | | 1 | | 2 |  |  | | |  | | |  | |
| X_44_ | | IW _Clutch-On,Gearbox-Neutral_ | |  | |  | 10 | 0.7 | | |  | | |  | |
| X_45_ | | LW _Clutch-On,Gearbox-Neutral_ | |  | |  |  |  | | | 1 | | |  | |
| X_46_ | | RW _Clutch-On,Gearbox-Neutral_ | | 1 | | 2 |  |  | | |  | | |  | |
| X_47_ | | IW _Gearbox-Neutral,PressGear1_ | |  | |  | 10 | 0.7 | | |  | | |  | |
| X_48_ | | LW _Gearbox-Neutral,PressGear1_ | |  | |  |  |  | | | 1 | | |  | |
| X_49_ | | RW _Gearbox-Neutral,PressGear1_ | | 1 | | 2 |  |  | | |  | | |  | |
| X_50_ | | IW _PressGear1,Gear1-On_ | |  | |  | 10 | 0.7 | | |  | | |  | |
| X_51_ | | LW _PressGear1,Gear1-On_ | |  | |  |  |  | | | 1 | | |  | |
| X_52_ | | RW _PressGear1,Gear1-On_ | | 1 | | 2 |  |  | | |  | | |  | |
| X_53_ | | IW _Gear1-On,PressAccelerator_ | |  | |  | 10 | 0.7 | | |  | | |  | |
| X_54_ | | LW _Gear1-On,PressAccelerator_ | |  | |  |  |  | | | 1 | | |  | |
| X_55_ | | RW _Gear1-On,PressAccelerator_ | | 1 | | 2 |  |  | | |  | | |  | |
| X_56_ | | IW _PressAccelerator,Accelerator-On_ | |  | |  | 10 | 0.7 | | |  | | |  | |
| X_57_ | | LW _PressAccelerator,Accelerator-On_ | |  | |  |  |  | | | 1 | | |  | |
| X_58_ | | RW _PressAccelerator,Accelerator-On_ | | 1 | | 2 |  |  | | |  | | |  | |
| X_59_ | | IW _Accelerator-On,Engine-On_ | |  | |  | 10 | 0.7 | | |  | | |  | |
| X_60_ | | LW _Accelerator-On,Engine-On_ | |  | |  |  |  | | | 1 | | |  | |
| X_61_ | | RW _Accelerator-On,Engine-On_ | | 1 | | 2 |  |  | | |  | | |  | |
| X_62_ | | IW _Engine-On,Rev-Meter-On_ | |  | |  | 10 | 0.7 | | |  | | |  | |
| X_63_ | | LW _Engine-On,Rev-Meter-On_ | |  | |  |  |  | | | 1 | | |  | |
| X_64_ | | RW _Engine-On,Rev-Meter-On_ | | 1 | | 2 |  |  | | |  | | |  | |
| X_65_ | | IW _RevMeter-On,BiteState_ | |  | |  | 10 | 0.7 | | |  | | |  | |
| X_66_ | | LW _RevMeter-On,BiteState_ | |  | |  |  |  | | | 1 | | |  | |
| X_67_ | | RW _RevMeter-On,BiteState_ | | 1 | | 2 |  |  | | |  | | |  | |
| X_68_ | | IW _Clutch-On,BiteState_ | |  | |  | 10 | 0.7 | | |  | | |  | |
| X_69_ | | LW _Clutch-On,BiteState_ | |  | |  |  |  | | | 1 | | |  | |
| X_70_ | | RW _Clutch-On,BiteState_ | | 1 | | 2 |  |  | | |  | | |  | |
| X_71_ | | IW _BiteState,PressAccelerator_ | |  | |  | 10 | 0.7 | | |  | | |  | |
| X_72_ | | LW _BiteState,PressAccelerator_ | |  | |  |  |  | | | 1 | | |  | |
| X_73_ | | RW _BiteState,PressAccelerator_ | | 1 | | 2 |  |  | | |  | | |  | |
| X_74_ | | IW _Engine-On,MovingState_ | |  | |  | 10 | 0.7 | | |  | | |  | |
| X_75_ | | LW _Engine-On,MovingState_ | |  | |  |  |  | | | 1 | | |  | |
| X_76_ | | RW _Engine-On,MovingState_ | | 1 | | 2 |  |  | | |  | | |  | |
| X_77_ | | IW _Gear1-On,MovingState_ | |  | |  | 10 | 0.7 | | |  | | |  | |
| X_78_ | | LW _Gear1-On,MovingState_ | |  | |  |  |  | | | 1 | | |  | |
| X_79_ | | RW _Gear1-On,MovingState_ | | 1 | | 2 |  |  | | |  | | |  | |
| X_80_ | | IS _Switch,TurnSwitch_ | | 1 | | 1 |  |  | | |  | | |  | |
| X_81_ | | IS _TurnSwitch,Engine-On_ | | 1 | | 1 |  |  | | |  | | |  | |
| X_82_ | | IS _Engine-On,FeelEngine-On_ | | 1 | | 1 |  |  | | |  | | |  | |
| X_83_ | | IS _FeelEngineOn,PressClutch_ | | 1 | | 1 |  |  | | |  | | |  | |
| X_84_ | | IS _PressClutch,Clutch-On_ | | 1 | | 1 |  |  | | |  | | |  | |
| X_85_ | | IS _Clutch-On,Gear-BoxNeutral_ | | 1 | | 1 |  |  | | |  | | |  | |
| X_86_ | | IS _GearBox-Neutral,PressGear1_ | | 1 | | 1 |  |  | | |  | | |  | |
| X_87_ | | IS _PressGear1,Gear1-On_ | | 1 | | 1 |  |  | | |  | | |  | |
| X_88_ | | IS _Gear1-On, PressAccelerator_ | | 1 | | 1 |  |  | | |  | | |  | |
| X_89_ | | IS _PressAccelerator, Accelerator-On_ | | 1 | | 1 |  |  | | |  | | |  | |
| X_90_ | | IS _Accelerator-On, Engine-On_ | | 1 | | 1 |  |  | | |  | | |  | |
| X_91_ | | IS _Engine-On,Rev-Meter-On_ | | 1 | | 1 |  |  | | |  | | |  | |
| X_92_ | | IS _RevMeter-On,BiteState_ | | 1 | | 1 |  |  | | |  | | |  | |
| X_93_ | | IS _Clutch-On,BiteState_ | | 1 | | 1 |  |  | | |  | | |  | |
| X_94_ | | IS _BiteState,PressAccelerator_ | | 1 | | 1 |  |  | | |  | | |  | |
| X_95_ | | IS _Engine-On,MovingState_ | | 1 | | 1 |  |  | | |  | | |  | |
| X_96_ | | IS _Gear-On1,MovingState_ | | 1 | | 1 |  |  | | |  | | |  | |
| X_97_ | | CIW _Switch,TurnSwitch_ | |  | |  | 10 | 0.4 | | |  | | |  | |
| X_98_ | | CIW _TurnSwitch,Engine-On_ | |  | |  | 10 | 0.4 | | |  | | |  | |
| X_99_ | | CIW _Engine-On,FeelEngine-On_ | |  | |  | 10 | 0.4 | | |  | | |  | |
| X_100_ | | CIW _FeelEngineOn,PressClutch_ | |  | |  | 10 | 0.4 | | |  | | |  | |
| X_101_ | | CIW _PressClutch,Clutch-On_ | |  | |  | 10 | 0.4 | | |  | | |  | |
| X_102_ | | CIW _Clutch-On,Gear-BoxNeutral_ | |  | |  | 10 | 0.4 | | |  | | |  | |
| X_103_ | | CIW _GearBox-Neutral,PressGear1_ | |  | |  | 10 | 0.4 | | |  | | |  | |
| X_104_ | | CIW _PressGear1,Gear1-On_ | |  | |  | 10 | 0.4 | | |  | | |  | |
| X_105_ | | CIW _Gear1-On, PressAccelerator_ | |  | |  | 10 | 0.4 | | |  | | |  | |
| X_106_ | | CIW _PressAccelerator, Accelerator-On_ | |  | |  | 10 | 0.4 | | |  | | |  | |
| X_107_ | | CIW _Accelerator-On, Engine-On_ | |  | |  | 10 | 0.4 | | |  | | |  | |
| X_108_ | | CIW _Engine-On,Rev-Meter-On_ | |  | |  | 10 | 0.4 | | |  | | |  | |
| X_109_ | | CIW _RevMeter-On,BiteState_ | |  | |  | 10 | 0.4 | | |  | | |  | |
| X_110_ | | CIW _Clutch-On,BiteState_ | |  | |  | 10 | 0.4 | | |  | | |  | |
| X_111_ | | CIW _BiteState,PressAccelerator_ | |  | |  | 10 | 0.4 | | |  | | |  | |
| X_112_ | | CIW _Engine-On,MovingState_ | |  | |  | 10 | 0.4 | | |  | | |  | |
| X_113_ | | CIW _Gear-On1,MovingState_ | |  | |  | 10 | 0.4 | | |  | | |  | |

| **iv initial values** | | **1** |
| --- | --- | --- |
| X_1_ | BS_Switch_ | 0 |
| X_2_ | BS_TurnSwitch_ | 0 |
| X_3_ | BS_Engine-0n_ | 0 |
| X_4_ | BS_FeelEngine-On_ | 0 |
| X_5_ | BS_PresClutch_ | 0 |
| X_6_ | BS_Clutch-On_ | 0 |
| X_7_ | BS_Gearbox-Neutral_ | 0 |
| X_8_ | BS_PressGear 1_ | 0 |
| X_9_ | BS_Gear1-On_ | 0 |
| X_10_ | BS_PressAccelerator_ | 0 |
| X_11_ | BS_Accelerator-On_ | 0 |
| X_12_ | BS_RevMeter-On_ | 0 |
| X_13_ | BSe_BiteState_ | 0 |
| X_14_ | BS_MovingState_ | 0 |
| X_15_ | OS_Switch_ | 1 |
| X_16_ | OS_TurnSwitch_ | 0 |
| X_17_ | OS_Engine-0n_ | 0 |
| X_18_ | OS_FeelEngine-On_ | 0 |
| X_19_ | OS_PressClutch_ | 0 |
| X_20_ | OS_Clutch-On_ | 0 |
| X_21_ | OS_Gearbox-Neutral_ | 0 |
| X_22_ | OS_PressGear 1_ | 0 |
| X_23_ | OS_Gear1_ | 0 |
| X_24_ | OS_PressAccelerator_ | 0 |
| X_25_ | OS_Accelerator-On_ | 0 |
| X_26_ | OS_RevMeter-On_ | 0 |
| X_27_ | OS_BiteState_ | 0 |
| X_28_ | OS_MovingState_ | 0 |
| X_29_ | IW _Switch,TurnSwitch_ | 0.1 |
| X_30_ | LW _Switch,TurnSwitch_ | 0.1 |
| X_31_ | RW _Switch,TurnSwitch_ | 0.1 |
| X_32_ | IW _TurnSwitch,Engine-On_ | 0.1 |
| X_33_ | LW _TurnSwitch,Engine-On_ | 0.1 |
| X_34_ | RW _TurnSwitch,Engine-On_ | 0.1 |
| X_35_ | IW _Engine-On,FeelEngine-On_ | 0.1 |
| X_36_ | LW _Engine-On,FeelEngine-On_ | 0.1 |
| X_37_ | RW _Engine-On,FeelEngine-On_ | 0.1 |
| X_38_ | IW _FeelEngine-On,PressClutch_ | 0.1 |
| X_39_ | LW _FeelEngine-On,PressClutch_ | 0.1 |
| X_40_ | RW _FeelEngine-On,PressClutch_ | 0.1 |
| X_41_ | IW _PressClutch,Clutch-On_ | 0.1 |
| X_42_ | LW _PressClutch,Clutch-On_ | 0.1 |
| X_43_ | RW _PressClutch,Clutch-On_ | 0.1 |
| X_44_ | IW _Clutch-On,Gearbox-Neutral_ | 0.1 |
| X_45_ | LW _Clutch-On,Gearbox-Neutral_ | 0.1 |
| X_46_ | RW _Clutch-On,Gearbox-Neutral_ | 0.1 |
| X_47_ | IW _Gearbox-Neutral,PressGear1_ | 0.1 |
| X_48_ | LW _Gearbox-Neutral,PressGear1_ | 0.1 |
| X_49_ | RW _Gearbox-Neutral,PressGear1_ | 0.1 |
| X_50_ | IW _PressGear1,Gear1-On_ | 0.1 |
| X_51_ | LW _PressGear1,Gear1-On_ | 0.1 |
| X_52_ | RW _PressGear1,Gear1-On_ | 0.1 |
| X_53_ | IW _Gear1-On,PressAccelerator_ | 0.1 |
| X_54_ | LW _Gear1-On,PressAccelerator_ | 0.1 |
| X_55_ | RW _Gear1-On,PressAccelerator_ | 0.1 |
| X_56_ | IW _PressAccelerator,Accelerator-On_ | 0.1 |
| X_57_ | LW _PressAccelerator,Accelerator-On_ | 0.1 |
| X_58_ | RW _PressAccelerator,Accelerator-On_ | 0.1 |
| X_59_ | IW _Accelerator-On,Engine-On_ | 0.1 |
| X_60_ | LW _Accelerator-On,Engine-On_ | 0.1 |
| X_61_ | RW _Accelerator-On,Engine-On_ | 0.1 |
| X_62_ | IW _Engine-On,Rev-Meter-On_ | 0.1 |
| X_63_ | LW _Engine-On,Rev-Meter-On_ | 0.1 |
| X_64_ | RW _Engine-On,Rev-Meter-On_ | 0.1 |
| X_65_ | IW _RevMeter-On,BiteState_ | 0.1 |
| X_66_ | LW _RevMeter-On,BiteState_ | 0.1 |
| X_67_ | RW _RevMeter-On,BiteState_ | 0.1 |
| X_68_ | IW _Clutch-On,BiteState_ | 0.1 |
| X_69_ | LW _Clutch-On,BiteState_ | 0.1 |
| X_70_ | RW _Clutch-On,BiteState_ | 0.1 |
| X_71_ | IW _BiteState,PressAccelerator_ | 0.1 |
| X_72_ | LW _BiteState,PressAccelerator_ | 0.1 |
| X_73_ | RW _BiteState,PressAccelerator_ | 0.1 |
| X_74_ | IW _Engine-On,MovingState_ | 0.1 |
| X_75_ | LW _Engine-On,MovingState_ | 0.1 |
| X_76_ | RW _Engine-On,MovingState_ | 0.1 |
| X_77_ | IW _Gear1-On,MovingState_ | 0.1 |
| X_78_ | LW _Gear1-On,MovingState_ | 0.1 |
| X_79_ | RW _Gear1-On,MovingState_ | 0.1 |
| X_80_ | IS _Switch,TurnSwitch_ | 1 |
| X_81_ | IS _TurnSwitch,Engine-On_ | 1 |
| X_82_ | IS _Engine-On,FeelEngine-On_ | 1 |
| X_83_ | IS _FeelEngineOn,PressClutch_ | 1 |
| X_84_ | IS _PressClutch,Clutch-On_ | 1 |
| X_85_ | IS _Clutch-On,Gear-BoxNeutral_ | 1 |
| X_86_ | IS _GearBox-Neutral,PressGear1_ | 1 |
| X_87_ | IS _PressGear1,Gear1-On_ | 1 |
| X_88_ | IS _Gear1-On, PressAccelerator_ | 1 |
| X_89_ | IS _PressAccelerator, Accelerator-On_ | 1 |
| X_90_ | IS _Accelerator-On, Engine-On_ | 1 |
| X_91_ | IS _Engine-On,Rev-Meter-On_ | 1 |
| X_92_ | IS _RevMeter-On,BiteState_ | 1 |
| X_93_ | IS _Clutch-On,BiteState_ | 1 |
| X_94_ | IS _BiteState,PressAccelerator_ | 1 |
| X_95_ | IS _Engine-On,MovingState_ | 1 |
| X_96_ | IS _Gear-On1,MovingState_ | 1 |
| X_97_ | CIW _Switch,TurnSwitch_ | 0 |
| X_98_ | CIW _TurnSwitch,Engine-On_ | 0 |
| X_99_ | CIW _Engine-On,FeelEngine-On_ | 0 |
| X_100_ | CIW _FeelEngineOn,PressClutch_ | 0 |
| X_101_ | CIW _PressClutch,Clutch-On_ | 0 |
| X_102_ | CIW _Clutch-On,Gear-BoxNeutral_ | 0 |
| X_103_ | CIW _GearBox-Neutral,PressGear1_ | 0 |
| X_104_ | CIW _PressGear1,Gear1-On_ | 0 |
| X_105_ | CIW _Gear1-On, PressAccelerator_ | 0 |
| X_106_ | CIW _PressAccelerator, Accelerator-On_ | 0 |
| X_107_ | CIW _Accelerator-On, Engine-On_ | 0 |
| X_108_ | CIW _Engine-On,Rev-Meter-On_ | 0 |
| X_109_ | CIW _RevMeter-On,BiteState_ | 0 |
| X_110_ | CIW _Clutch-On,BiteState_ | 0 |
| X_111_ | CIW _BiteState,PressAccelerator_ | 0 |
| X_112_ | CIW _Engine-On,MovingState_ | 0 |
| X_113_ | CIW _Gear-On1,MovingState_ | 0 |
